# Aldo-keto reductases protect metastatic melanoma from ER stress-independent ferroptosis

**DOI:** 10.1038/s41419-019-2143-7

**Published:** 2019-11-28

**Authors:** Mara Gagliardi, Diego Cotella, Claudio Santoro, Davide Corà, Nickolai A. Barlev, Mauro Piacentini, Marco Corazzari

**Affiliations:** 1 Department of Biology, University of Rome Tor Vergata, Rome, Italy; 2 Department of Health Sciences, University of Piemonte Orientale, Novara, Italy; 3 Center for Translational Research on Autoimmune and Allergic Disease (CAAD), University of Piemonte Orientale, Novara, Italy; 4 Department of Health Sciences and Interdisciplinary Research Center of Autoimmune Diseases (IRCAD), University of Piemonte Orientale, Novara, Italy; 5 Department of Translational Medicine, University of Piemonte Orientale, Novara, Italy; 6 Laboratory of Molecular Medicine, Institute of Cytology of the Russian Academy of Sciences, Saint Petersburg, Russia; 7 Laboratory of Intracellular Signaling, Moscow Institute of Physics and Technology, Dolgoprudny, Moscow Region Russian Federation

**Keywords:** Melanoma, Cell death

## Abstract

The incidence of melanoma is increasing over the years with a still poor prognosis and the lack of a cure able to guarantee an adequate survival of patients. Although the new immuno-based coupled to target therapeutic strategy is encouraging, the appearance of targeted/cross-resistance and/or side effects such as autoimmune disorders could limit its clinical use. Alternative therapeutic strategies are therefore urgently needed to efficiently kill melanoma cells. Ferroptosis induction and execution were evaluated in metastasis-derived wild-type and oncogenic BRAF melanoma cells, and the process responsible for the resistance has been dissected at molecular level. Although efficiently induced in all cells, in an oncogenic BRAF- and ER stress-independent way, most cells were resistant to ferroptosis execution. At molecular level we found that: resistant cells efficiently activate NRF2 which in turn upregulates the early ferroptotic marker CHAC1, in an ER stress-independent manner, and the aldo-keto reductases AKR1C1 ÷ 3 which degrades the 12/15-LOX-generated lipid peroxides thus resulting in ferroptotic cell death resistance. However, inhibiting AKRs activity/expression completely resensitizes resistant melanoma cells to ferroptosis execution. Finally, we found that the ferroptotic susceptibility associated with the differentiation of melanoma cells cannot be applied to metastatic-derived cells, due to the EMT-associated gene expression reprogramming process. However, we identified SCL7A11 as a valuable marker to predict the susceptibility of metastatic melanoma cells to ferroptosis. Our results identify the use of pro-ferroptotic drugs coupled to AKRs inhibitors as a new valuable strategy to efficiently kill human skin melanoma cells.

## Introduction

Cutaneous melanoma represents one of the most aggressive and difficult to treat forms of human cancer, with a worldwide incidence that has steadily increased over the past 40 years^1,2^. Notoriously unresponsive to conventional chemotherapy, the metastatic disease is highly invasive and evolves with an extensive repertoire of molecular defenses against immunological and cytotoxic attacks^3^. Although linked to exposure to ultraviolet light, both genotypic and phenotypic changes in melanocytes predispose to transformation and melanomagenesis^4,5^. Although several gene mutations have been observed in this malignancy^6^, oncogenic mutations in the Ras/Raf pathway are those most frequently associated with the development of melanoma^7^. Indeed, up to 80% of all melanomas harbor activating NRAS or BRAF mutations^8,9^, with BRAF^V600E^ representing more than 90% of BRAF mutations, resulting in constitutive activation of the signaling pathway, promoting melanoma proliferation and resistance to apoptosis^10^. Since both NRAS/BRAF mutations are frequently present in benign nevi, other factors are required to drive melanomagenesis^7,8^. In fact, both autophagy and ER stress, primarily pro-survival processes, have accordingly been postulated as secondary events contributing to melanoma development and, importantly, playing a key role in chemoresistance^11,12^. Particularly, oncogenic BRAF drives a chronic ER stress status directly controlling basal autophagy^13,14^, resulting in enhanced chemoresistance of these cells^13,15^. In contrast, the pharmacological alteration of autophagy has a positive effect on the response of BRAF wild-type melanomas to treatment^14^, indicating the high heterogeneity of this malignancy with the ability to evolve with an extensive repertoire of molecular defenses against pro-death stimuli.

In the past decade, treatment options for metastatic melanoma have significantly increased with the arrival of BRAF inhibitors and BRAF/MEK combination therapy. However, despite FDA-approved BRAF-targeted therapies for advanced stage melanoma showed a great deal of promise, development of rapid resistance limits their success^16–18^. Therefore, the success of melanoma therapy still remains one of the worst, compared to other malignancies. Very recently, an immune-based therapeutic approach has been developed, mainly using CTLA-4 and/or PD-1 inhibitor. PD-1 inhibitors seem to be the most promising as first-line therapy as they can produce high response rates with long-term tumor remission, while maintaining more tolerable side effect profiles^19–22^. Combinations of targeted therapies and immune-based therapies may show improved efficacy, but also carry an increased risk of adverse effects, together with cross-resistance^23^. Therefore, novel therapeutic strategies are urgently needed.

Recently, an alternative iron-dependent cell death pathway, named ferroptosis, has been identified^24^. Although the precise molecular mechanisms regulating this death pathway are still elusive and under deep investigation, its induction was originally described relying on the inhibition of the cystine uptake by the cystine/glutamate antiporter system (system XC^−^), located onto the cell membrane^24^, resulting in the inhibition of glutathione peroxidase 4 (Gpx4) and depletion of glutathione, determining the imbalance of the reduced/oxidized glutathione system, the main antioxidant cell system, and induction of the ER stress pathway^25^. Downstream, lipid peroxides generation represents a key step mediating the execution of this death process^26^. In this context, the downstream upregulation/activation of specific members of the aldo-keto reductases (AKRs) gene family (AKR1C1 ÷ 3) has been proposed as factors catalyzing the conversion of aldehydes and ketones to their corresponding alcohols, therefore possibly detoxifying the highly dangerous reactive molecules lipid peroxides, thus inhibiting the execution of ferroptosis^24^.

Here we show that ferroptosis is potentially inducible in melanoma cell lines although its execution is inhibited in most of them, due to the upregulation and activation of downstream AKRs, through the main oxidative stress-linked transcription factor NRF2. However, we demonstrate that inhibiting the expression/activity of these enzymes or blocking NRF2 action re-establish the normal execution of ferroptosis.

## Results

### Erastin induces an ER stress-independent upregulation of the early ferroptotic marker CHAC1 in melanoma cells

**Fig. 1:**
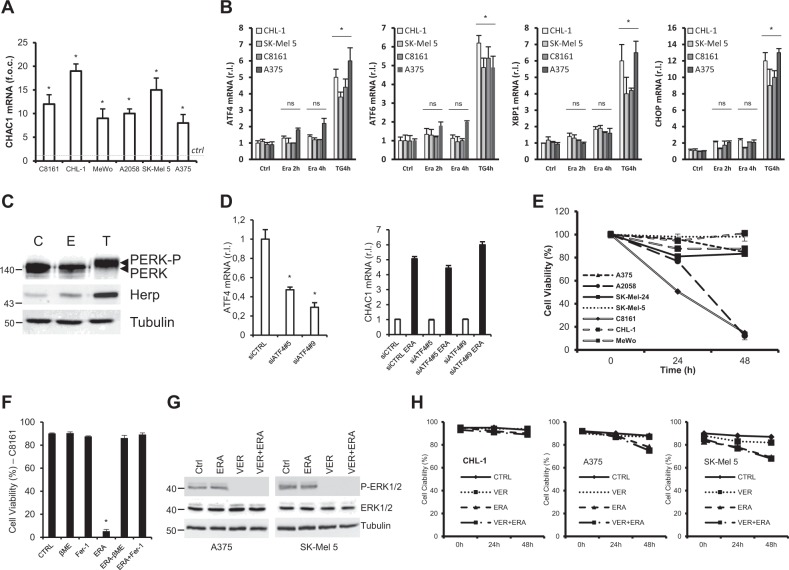
Ferroptosis induction and execution in melanoma cells is ER stress- and oncogenic BRAF- independent. **a** The indicated melanoma cell lines were treated 2 h with erastin (10 μM) and CHAC1 expression was evaluated by qRT-PCR. (The histograms represent the expression of CHAC1 stimulated by erastin with respect to each control, which has been arbitrarily set on 1, in each individual cell line, *dotted line*). **b** The expression level of the ER stress markers ATF4, ATF6, XBP1, and CHOP was evaluated in CHL-1, C8161, SK-Mel 5 and A375 cell lines treated or untreated with erastin, at indicated time points, by qRT-PCR. Thapsigargin (10 μg/ml, 4 h) was used as positive control. **c** The activation of PERK and Herp upregulation were evaluated in CHL-1 cells treated (2 h) or untreated, C, with erastin, E, or thapsigargin, T, by western blotting analysis. Tubulin was used as loading control. **d** The expression of ATF4 was downregulated in CHL-1 cells by transient transfection of specific siRNA sequences (siATF4#5 and siATF4#9; a scrambled sequence was used as control, shCTRL) and ATF4 (left panel) or CHAC1 (right panel) mRNA levels were evaluated by qRT-PCR, in cells treated or untreated 2 h with erastin (10 μM), as indicated. **e** The indicated melanoma cell lines were exposed 24 or 48 h to erastin (10 μM) and cell viability was evaluated by measuring the percentage of FDA^+^/7AAD^-^ cells, by flow cytometry. **f** C8161 cells were exposed to ERA (10 μM) in presence or absence of 2-Mercaptoethanol (βME, 100μM) or Ferrostatin-1 (Fer-1, 10μM) and cell viability was evaluated 48 h post-treatment, as reported in E. **g** A375 and SK-Mel 5 cells were treated 8 h with erastin (10 μM) in presence or absence of Vemurafenib (VER, 10 μM; 2 h pre-treatment) and BRAF kinase activity was evaluated by measuring the phosphorylation levels of the protein target ERK1/2 (P-ERK1/2). Tubulin and ERK1/2 were used as loading controls. **h** CHL-1, A375 or SK-Mel 5 cells were exposed to ERA (10 μM; 24 or 48 h) in presence or absence of Vemurafenib (VER, 10 μM; 2 h pre-treatment) and cell viability was evaluated as in E. (Histograms represent mean ± s.d.; *n* = 3; ns = not significant, compared to controls; *p < 0,05, compared to controls; w.b.; images are representative of three independent experiments).

Although the precise molecular mechanisms of ferroptosis are still unknown and under deep investigation, the upregulation of CHAC1 is widely accepted as an early ferroptotic marker, also contributing to glutathione degradation and ferroptosis execution^24^. Therefore, we decided to evaluate the ability of the typical inducer erastin (ERA) to stimulate the ferroptotic induction in melanoma cells. To this aim, we exposed our panel of wild-type and oncogenic BRAF human melanoma cell lines^13^ to ERA and CHAC1 gene expression was evaluated by qRT-PCR. Data shown in Fig. 1a indicate that the expression of CHAC1 is promptly upregulated in all tested melanoma cells (2 h post-ERA treatment). Moreover, enhanced CHAC1 expression levels are maintained even later, indicating the progress of the ferroptotic process (Suppl. S1). Since previous reports linked the expression of CHAC1 to ER stress induction^27^ with the latter signaling pathway potentially contributing to ferroptosis^25^, we evaluated whether this was also the case with melanoma cells. To explore this hypothesis, CHL-1, A375, C8161, and SK-Mel 5 cells were treated or untreated 2 and 4 h with ERA and the expression of the ER stress markers ATF4, ATF6, XBP1 and CHOP/Gadd153 was evaluated by qRT-PCR, using thapsigargin (TG) as positive control. Surprisingly, our results indicate no significant upregulation of any of the ER stress marker (Fig. 1b), indicating that ER stress is not involved in ferroptosis induction and CHAC1 upregulation is ER stress-independent under pro-ferroptotic stimuli, at least in melanoma cells. To further support these conclusions, we also dissected out the activity of the ER stress PERK/ATF4/CHOP signaling pathway, previously indicated to be responsible for CHAC1 gene expression regulation^28^. To this aim, CHL-1 cells were treated or untreated 2 h with ERA or TG and the phosphorylation of PERK and the downstream ERAD marker Herp^29^ were evaluated by western blotting analysis^30^. Data reported in Fig. 1c clearly show neither the activation of PERK (upper panel), nor a significant upregulation of Herp (bottom panel). We also inhibited the expression of ATF4 in CHL-1 cells by transiently transfecting two siRNA oligos specific for ATF4 (siATF4#5 and siATF4#9), using a scrambled sequence (siCTRL) as a control (Fig. 1d, left panel), and evaluated the expression of CHAC1 under ERA treatment (2 h), by qRT-PCR. Results indicate the ablation of ATF4 expression does not prevent the ERA-stimulated upregulation of CHAC1 (Fig. 1d, right panel). Similar results were also obtained inhibiting the expression of CHOP, in the same experimental conditions (Suppl. S2). Collectively these results indicate that erastin induces the early expression of the ferroptotic marker CHAC1 in an ER stress-independent manner, thus also indicating that the latter process is not evoked during ferroptosis stimulation in melanoma cells.

### Most melanoma cell lines are resistant to ferroptosis execution, independently from BRAF oncogenic status

To verify the efficacy of ferroptosis induction resulting in melanoma cell death completion, the cell viability of our panel of melanoma cells was evaluated at 24 and 48 h post-erastin treatment. Surprisingly, the data shown in Fig. 1e (and Suppl. S3) indicate that most (five out of seven) of melanoma cell lines are resistant to ferroptosis execution despite early CHAC1 upregulation (Fig. 1a). To confirm that erastin was able to stimulate the ferroptotic process in sensitive cell lines, C8161 cells were then exposed to ERA in presence or absence of the ferroptotic inhibitors Ferrostatin-1^24^ or 2-Mercaptoethanol^25^ and cell viability was evaluated. Results shown in Fig. 1f (and Suppl. S3) demonstrate that ERA-induced cell death was completely inhibited by Ferrostatin-1 or 2-Mercaptoethanol, indicating that C8161 cells were dying through ferroptosis. Importantly, data shown in Fig. 1e also indicate that melanoma cell resistance to ferroptosis execution is potentially independent of BRAF mutational status, since oncogenic BRAF cells were both sensitive (A2058 and C8161) or resistant (A375, SK-Mel 5) to ferroptosis execution. To further exclude a role of BRAF mutations in melanoma resistance to ferroptotic cell death, A375 and SK-Mel 5 oncogenic BRAF melanoma cell lines were treated with erastin in presence or absence of the specific BRAF inhibitor Vemurafenib (VER, 10 μM; 2 h pre-treatment), using the BRAF^WT^ CHL-1 cell line as negative control. Our results indicate that inhibiting the activity of mutant BRAF (Fig. 1g) does not resensitize melanoma cells to ferroptosis execution (Fig. 1h), at least as short-term treatment.

### Ferroptosis resistance is associated with downstream AKR1C1 ÷ 3 upregulation

**Fig. 2:**
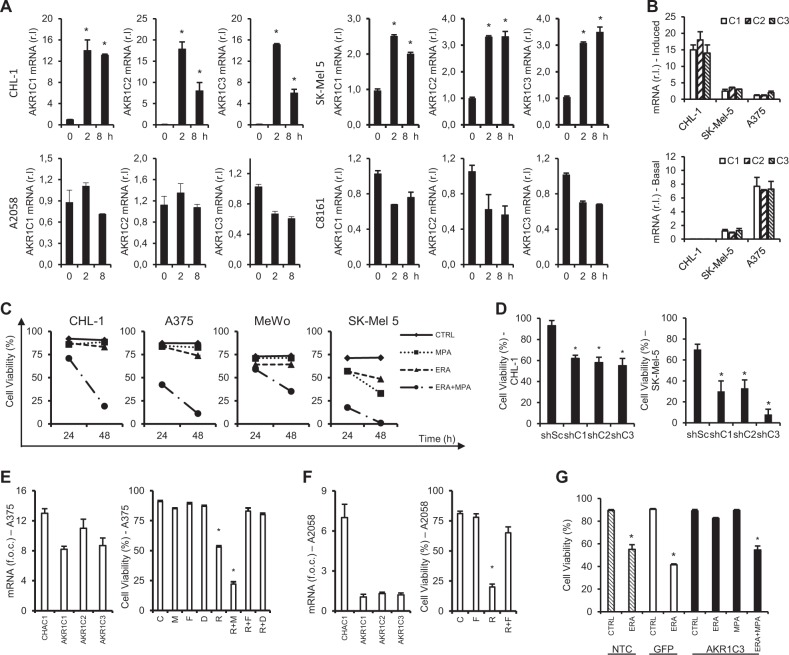
AKR1C1 ÷ 3 confer resistance to ferroptosis execution. **a** CHL-1, SK-Mel 5, A2058 and C8161 cells were treated or untreated 2 or 8 h with ERA (10 μM) and mRNA level of each AKR1C was evaluated by qRT-PCR. **b** CHL-1, SK-Mel 5 and A375 were exposed 2 h to ERA (10 μM), the expression of each AKR1C was evaluated by qRT-PCR and a comparative analysis among the three cell line was performed to highlight the relative AKR1Cs induced expression (upper panel); the expression of each AKR1C was also evaluated in CHL-1, SK-Mel 5 and A375 in resting conditions, by qRT-PCR, and a comparative analysis among the three cell line was performed, to highlight the relative AKR1Cs basal expression (bottom panel). **c** CHL-1, A375, MeWo and SK-Mel 5 cells were untreated (CTRL) or treated with ERA (10 μM) or MPA (10 μM) alone or in combination (ERA + MPA), and cell viability was evaluated at 24 or 48 h by flow cytometry. **d** CHL-1 or SK-Mel 5 were infected with lentiviral particles carrying specific shRNA targeting AKR1C1 (shC1), AKR1C2 (shC2) or AKR1C3 (shC3)—a scrambled sequence was used as control (shSc)—and cells were exposed to ERA (10μM) for 24 h. The expression of each AKR was evaluated 48 h post-infection by qRT-PCR (Suppl S6), and cell viability was quantified by flow cytometry. **e** A375 cells were untreated, C, or treated with RLS3 (2 μM; R) alone or in combination with MPA (10 μM; M), Ferrostatin-1 (10μM; F), or Deferoxamine (100 μM; D), and cell viability was evaluated 24 h post-treatment by flow cytometry (right panel), or CHAC1 and AKR1C1 ÷ 3 expression was evaluated 2 h post-treatment, by qRT-PCR (left panel). **f** A2058 cells were untreated, C, or treated with RLS3 (2μM; R) alone or in combination Ferrostatin-1 (10 μM; F), and cell viability was evaluated 24 h post-treatment by flow cytometry (right panel), or CHAC1 and AKR1C1 ÷ 3 expression was evaluated 2 h post-treatment, by qRT-PCR (left panel). (Histograms represent mean ± s.d.; *n* = 3; *p < 0,05). **g** A2058 cells were not transfected (NTC) or transiently transfected with a vector coding for human AKR1C3, or a GFP encoding vector (used as control). Next, cells were untreated (CTRL) or exposed 24 h to 10μM erastin (ERA) alone or in combination with 10 μM MPA. Cell viability was than evaluated by flow cytometry (Histograms are representative of mean ± s.d.; *n* = 3; *p < 0,05).

Cancer cells are characterized by deregulated proliferation and evolve a wide repertoire of defense mechanisms promoting cell survival and inhibiting cell death, particularly active under adverse environmental conditions such as anti-cancer treatments. Therefore, it is not surprising that many melanoma cells can also be resistant to ferroptosis. Understanding the molecular mechanism(s) conferring resistance might therefore be useful to identify new therapeutic targets. To this aim, we took advantage of results from Stockwell work indicating that AKRs are potentially downstream ferroptotic inhibitors^24^. AKRs are a family of enzymes basically deputed to the conversion of aldehydes or ketones into their corresponding alcohols, thus inhibiting their cytotoxic potential. They are implicated in several biochemical pathways and especially abundant in the liver^31^. Their anti-ferroptotic role is thus potentially related to their detoxifying activity, resulting in the reduction of lipid peroxides, the key executioners of the ferroptotic cell death process^32^. To explore whether this was the case in melanoma, we evaluated the mRNA levels of the three candidates AKR1C1, AKR1C2, and AKR1C3 in our cell lines, upon erastin treatment. Therefore, resistant CHL-1 and SK-Mel 5 were exposed 2 or 8 h to 10 μM ERA and mRNA levels of the three enzymes were evaluated by qRT-PCR. Results were then compared to those from the sensitive C8161 and A2058 cell lines, obtained in the same experimental conditions. Data indicate that the expression of all three enzymes was promptly and significantly upregulated in both resistant cell lines (CHL-1 and SK-Mel 5) to as early as 2 h after treatment. In contrast, no upregulation—but an interestingly slight but consistent downregulation—was observed in the sensitive (C8161 and A2058) cell lines of any of the three enzymes (Fig. 2a). These results were confirmed by western blotting analysis, in which increased protein levels of AKR1C1 ÷ 3 were evident in resistant A375 cells treated 16 h with erastin, compared to control, while no upregulation was observed in the sensitive C8161 (Suppl. S4). Collectively these data indicate a potential role of AKRs upregulation in melanoma cell resistance to ferroptosis execution. Due to the high heterogeneity of AKR1C1 ÷ 3 upregulation under erastin treatment observed in resistant cells, we next compared both basal and stimulated (2 h ERA) mRNA levels of each individual AKR1Cs among resistant cell lines CHL-1, SK-Mel 5, and A375. Interestingly, this analysis revealed, with good approximation, an inversely proportional relationship between basal (Fig. 2b, bottom panel) and stimulated expression (Fig. 2b, upper panel), of each of the three genes and among the three cell lines.

### AKRs inhibition resensitize cells to ferroptosis execution

To verify the role of AKRs enzymatic activity in lipid peroxide demolition in melanoma cells, resistant cell lines were exposed 24 or 48 h to ERA, in presence or absence of the AKR1C1 ÷ 3 pan inhibitor medroxyprogesterone^33^ (MPA; 10 μM), and cell viability was evaluated as reported above. Data shown in Fig. 2c and supplementary S3 and S5 clearly indicate that combined ERA + MPA treatment consistently resensitize all resistant melanoma cell lines to ferroptotic cell death, although with different kinetics. Next, we individually inhibited the expression of each of the three enzymes in both CHL-1 and SK-Mel 5 by infecting cells with lentiviral particles carrying specific shRNA targeting the three targets. A total of five shRNA sequences were tested for each individual AKR and the one conferring the best silencing efficiency was used in the next experiments (shC1 to AKR1C1, shC2 to AKR1C2, and shC3 to AKR1C3); a scrambled shRNA sequence was used as control (shSc). The silencing efficiency was confirmed 48 h post-infection by qRT-PCR analysis (Suppl. S6), cells were then exposed 24 h to erastin and cell viability was evaluated by flow cytometry. Data reported in Fig. 2d indicate that inhibiting the expression of each individual enzyme results in enhanced erastin-stimulated ferroptotic cell death in both cell lines. To further confirm these results, A375 and A2058 cells were treated with a second ferroptotic inducer, RLS3. Thus, both cell lines were exposed to 2 μM RLS3 and both AKR1C1 ÷ 3 and CHAC1 gene expression were evaluated 2 h post-treatment, by qRT-PCR. As expected, CHAC1 expression was upregulated in both cell lines, thus confirming results reported in Fig. 1a, while AKR1C1 ÷ 3 mRNA levels were increased in A375 (Fig. 2e, left panel) but not in A2058 cells (Fig. 2f, left panel). The same cell lines were then exposed 24 h to RLS3 alone or in combination with Ferrostatin-1 (both cell lines) or with MPA or Deferoxamine (an iron chelator;^24^ A375 only), and cell viability was evaluated by flow cytometry. Data reported in Fig. 2e and F (right panels) show that A2058 were sensitive to RLS3 induced cell death, while A375 were mostly resistant; moreover, RLS3 treatment induces a ferroptotic cell death process in both cell lines since ferrostatin-1 (A375 and A2058) or deferoxamine (A375) completely protected both cell lines (Fig. 2e, f). Importantly, data reported in Fig. 2e, f also show a clear correlation between cell resistance to pro-ferroptotic drug and AKR1C1 ÷ 3 gene expression upregulation, thus further sustaining previous conclusions. Finally, AKR1C3 was transiently over-expressed in A2058, by transient transfection (Suppl. S7), and cell viability was evaluated 24 h post-erastin treatment, in presence or absence of MPA (Fig. 2g). Data reported in Fig. 2g and Supplementary S7 clearly show that AKR1C3 overexpression confers resistance of sensitive A2058 to erastin-induced ferroptotic cell death while MPA, inhibiting AKR1C3 enzymatic activity, reverts the process.

Collectively these results show that AKR1C1 ÷ 3 gene expression upregulation and enzymatic activity are responsible for inhibited ferroptosis execution in resistant melanoma cells and that the activity of each individual enzyme seems to be equally required.

### LOX-mediated lipid peroxides generation are essential for ferroptosis execution

**Fig. 3:**
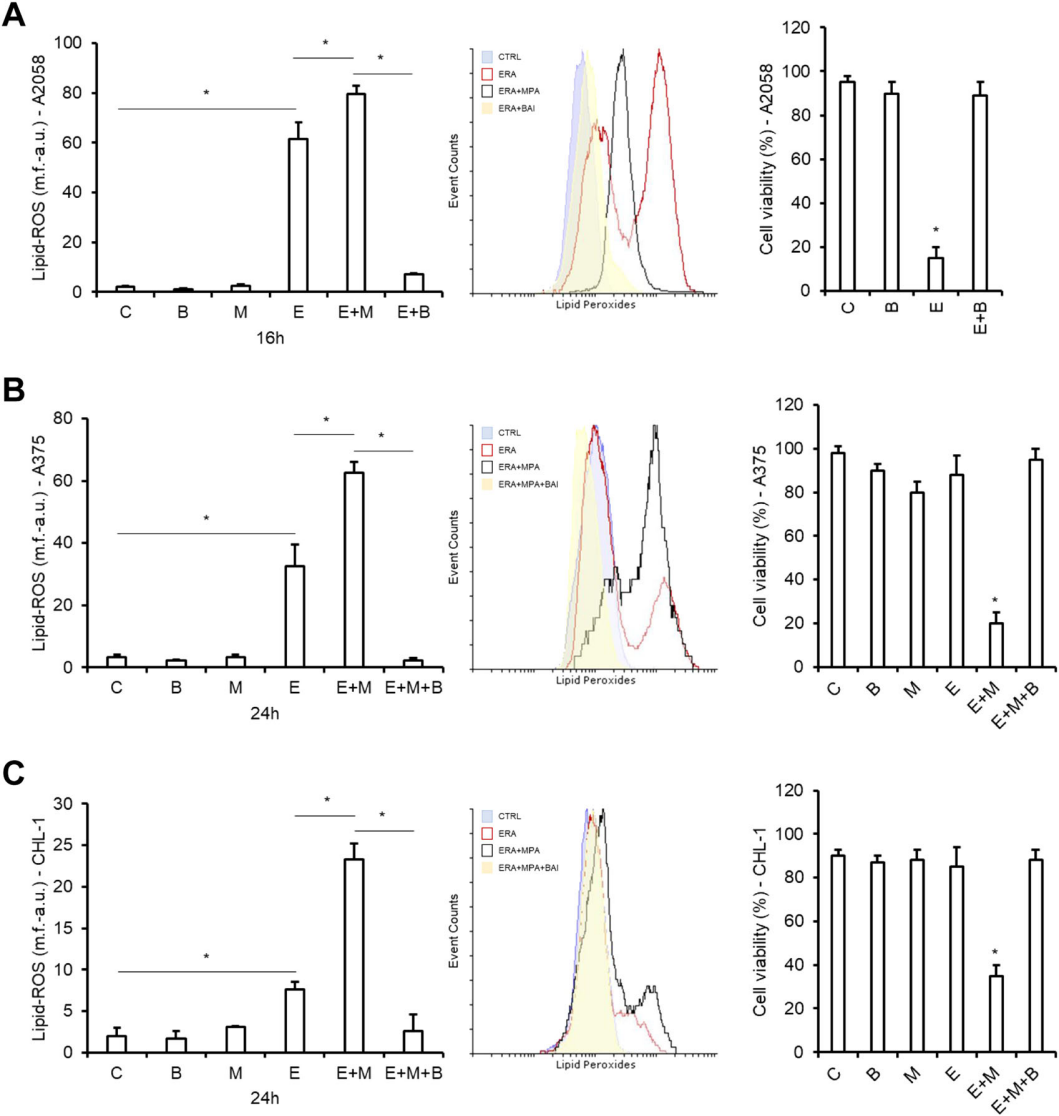
Lipid peroxides generation by 12/15-LOX and degradation by AKR1C1÷3. A2058 (**a**), A375 (**b**) and CHL-1 (**c**) were untreated “C”, or treated or with 20 μM Baicalein “B”, 10 μM MPA “M”, 10 μM Erastin “E”, Erastin plus MPA “E + M”, Erastin plus Baicalein (A2058) “E + B” or Erastin plus MPA plus Baicalein (A375 and CHL-1) “E + M + B”. Lipid peroxides generation was evaluated at 16 h (A2058) or 24 h (A375 and CHL-1) (left panels), while cell viability was evaluated at 24 h (A2058) or 48 h (A375 and CHL-1) (right panels). Representative flow cytometry profiles of lipid ROS staining are also shown. (Histograms represent mean ± s.d.; *n* = 3; *p < 0,05).

Our above results showing AKR1C1 ÷ 3 enzymatic activity resulting in ferroptosis execution inhibition imply that lipid peroxides production are crucial executioners of the ferroptotic cell death program also in melanoma cells. Indeed, although the detailed molecular mechanism(s) driving the ferroptotic cell death process from induction to execution is still unclear, the key role played by lipid peroxides is, however, well consolidated although the link between their generation and action is still not completely known^32,34^. Interestingly, although their generation was initially considered the result of intracellular iron accumulation and consequent Fenton’s reactions^35^, it is now evident that they can also be produced by intracellular lipoxygenases enzymatic activity^36^. Therefore, to evaluate the role and source of lipid peroxides in our system, sensitive A2058 cells were exposed 16 h to erastin and lipid peroxides generation was evaluated by measuring the fluorescence of the specific probe BODIPY C11, by flow cytometry. Results reported in Fig. 3a (left and middle panels) show a huge generation of lipid peroxides under erastin treatment (compare E with C columns, erastin and control respectively), thus preceding the cell death execution. Interestingly, while A375 and CHL-1 cells were treated as the sensitive A2058, a consistently lower lipid peroxides production was evidenced in both cell lines, even at 24 h post-treatment (Fig. 3b, c, left and middle panels), perfectly correlating with enhanced AKR1Cs expression and resistance of these cell lines to erastin-induced ferroptotic cell death (Figs. 2a and 1e, respectively). In fact, the link between erastin-induced lipid peroxides levels and ferroptosis execution is clearly shown by the enhanced elevation of lipid peroxides in cells exposed to ERA in presence of MPA, compared to ERA alone (compare E + M with E columns in left panels of Fig. 3a–c), resulting in cell death induction in both resistant cell lines (Fig. 3b, c, right panels, compare E with E + M columns). To determine the source of lipid peroxides generation, we exposed our sensitive A2058 cell line to erastin in the presence or absence of the 12/15-lipoxygenase (ALOX12 and ALOX15) inhibitor Baicalein (BAI)^37^ and lipid peroxides generation and cell death were evaluated. Data reported in Fig. 3a show that BAI completely prevented both lipid peroxides generation and ferroptosis execution in A2058 exposed to ERA. Next, we exposed the two resistant A375 and CHL-1 cells to erastin in presence of MPA—to inhibit AKR1Cs activity and lipid peroxides degradation—or Baicalein—to inhibit 12/15-LOX—and lipid peroxides and cell viability were evaluated at 24 and 48 h, respectively. Data reported in Fig. 3b, c show that BAI completely inhibited both lipid peroxides generation and ferroptotic cell death induction, in both cell lines in which the AKR1Cs activity was inhibited by MPA (compare E + M with E + M + B columns). These results are also confirmed by data showing that BAI completely abrogated the accumulation of lipid peroxides in A375 cells exposed to RLS3, while Zileuton—a 5-lipoxygenase inhibitor—did not (Suppl. S8). Collectively these data indicate that lipid peroxides are key executioners of the ferroptotic program also in melanoma cells, are generated by 12/15-lipoxygenase and degraded by AKR1C1 ÷ 3.

### NRF2 regulates AKR1C1 ÷ 3 expression upon ferroptosis induction

**Fig. 4:**
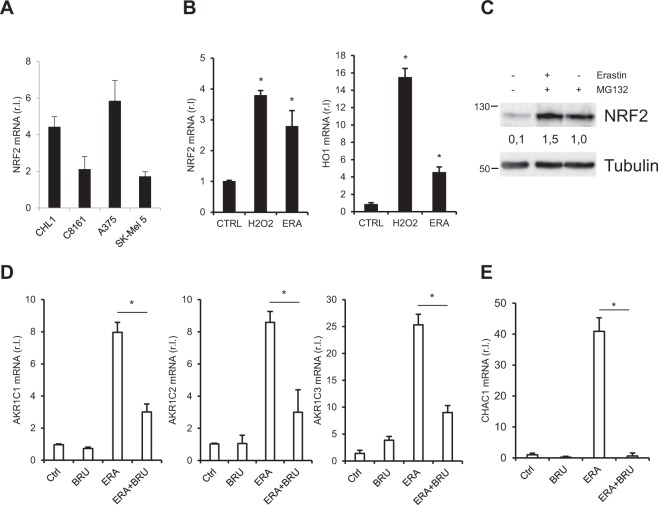
NRF2 is involved in ferroptosis resistance of melanoma cells. **a** NRF2 basal expression was evaluated in CHL-1, C8161, A375 and SK-Mel 5 cells by qRT-PCR. **b** CHL-1 cells were treated or untreated with 10μM erastin or 500 μM H_2_O_2_ and NRF2 (left panel) or HO1 (right panel) mRNA levels were evaluated by qRT-PCR. **c** NRF2 protein levels were measured in CHL-1 cells exposed to 10μM erastin in presence or absence of 10μM MG132 (2 h pre-treatment); densitometric analysis was reported. CHL-1 cells were exposed to 10μM erastin, 50 nM Brusatol individually or concomitantly (ERA + BRU) and AKR1C1 ÷ 3 (**d**) or CHAC1 (**e**) mRNA levels were evaluated by qRT-PCR. (Histograms represent mean ± s.d.; *n* = 3; *p < 0,05; w.b. images are representative of three independent experiments).

Due to the key role played by AKRs in ferroptosis resistance in melanoma cells, we investigated the molecular mechanism(s) regulating their expression. To this aim, we focused our attention on the canonical transcription factor (TF) NRF2, representing a perfect link between oxidative stress response^38^ and AKRs regulation^39^. Since NRF2 is usually dysregulated in cancer^40^, including melanoma^41^, we first evaluated the transcriptional levels of this TF in our cell lines. As shown in Fig. 4a, data confirm a heterogeneous expression of NRF2 in melanoma cells. Next, we evaluated the potential activation of this TF upon ferroptosis induction. To explore this hypothesis, resistant and sensitive cells were exposed to ERA (4 h) or H_2_O_2_ (positive control), and mRNA levels of NRF2 or its target HO1 were evaluated by qRT-PCR (Fig. 4b, c and Suppl. S9A). Data indicated the upregulation of NRF2 under ERA treatment, paralleled by enhanced expression of its downstream gene target HO1 (Fig. 4b right panel). To further confirm these results, NRF2 protein levels were evaluated in the same cells treated or untreated with erastin (4 h) in presence of MG132 (2 h pre-treatment), to inhibit NRF2 proteasome-mediated degradation. Western blotting analysis reported in Fig. 4c and Supplementary S9B show enhanced protein levels in cells co-treated with ERA and MG132 compared to MG132 alone. Collectively these data indicate that NRF2 is activated early during ferroptosis induction. Next, to evaluate the potential contribution of NRF2 activation in the ERA-stimulated AKR1C1 ÷ 3 upregulation, CHL-1 cells were treated or untreated with ERA (4 h) in presence or absence of Brusatol^42^, an NRF2 inhibitor, and mRNA levels of the three AKR1Cs were evaluated by qRT-PCR. Results clearly indicate that inhibiting NRF2 activity results in consistent reduction of erastin-mediated AKR1C1 ÷ 3 upregulation (Fig. 4d). Finally, to check whether NRF2 activation under ferroptotic condition might also be responsible for the ER stress-independent CHAC1 early upregulation, the mRNA levels of CHAC1 were evaluated in the same experimental conditions, in both sensitive and resistant cell lines. Data reported in Fig. 4e and Supplementary S9C indicate that NRF2 inhibition resulted in complete abrogation of ERA-stimulated CHAC1 upregulation. These data are also confirmed by results showing enhanced basal expression of both CHAC1 (3,02) and AKR1C1 (1,75) in CHL-1 cells over-expressing NRF2, compared to control cells (GFP; Suppl. S10).

### Melanoma cell lines differentiation and ferroptosis resistance

**Fig. 5:**
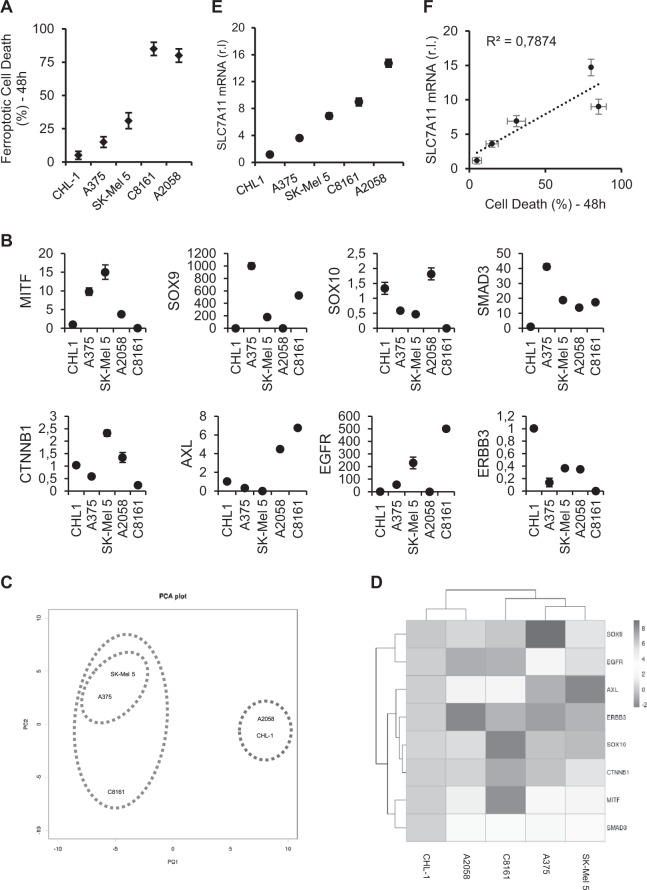
Melanoma cells differentiation and ferroptosis resistance. CHL-1, A375, SK-Mel 5, C8161 and A2058 were treated 48 h with 10μM ERA and cell viability was evaluated by flow cytometry (**a**). The basal expression of indicated genes (Transcription Factors: MITF, SOX9, SOX10, SMAD3, CTNBB1 and RTKs: AXL, EGFR, ERBB3) was evaluated in the same cell lines in A, by qRT-PCR, using CHL-1 as reference (which expression of each gene was set to 1; these data are representative of 4 independent experiments. **b** A Principal Component Analysis (PCA, (**c**) and hierarchical cluster analysis (**d**) was performed by using gene expression analysis data reported in B. The basal expression of SLC7A11 was evaluated in the same cell lines in A, by qRT-PCR, using CHL-1 as reference (which expression was set to 1. **e**. SLC7A11 basal expression and the % (reported in A) of cell death of each cell line were plotted together (scatter plot) and the correlation coefficient R^2^ was calculated (**f**). (*n* = 3; each point represents mean ± s.d.).

During the preparation of this manuscript, Graeber’s research group published data showing that primary tumor-derived melanoma cells are characterized by a wide ‘differentiation status’, sharing a gene expression signature based on a panel of nine genes^43^. Interestingly, they identified a correlation between cancer cell differentiation and ferroptotic cell death susceptibility. Since our experimental model is based on metastasis-derived melanoma cells, with no specific and available indication about their differentiation status, we decided to evaluate and compare the gene expression level of Graeber’s identified ‘differentiation genes panel’ in our panel of cell lines and verify a potential correlation with sensitivity to erastin-induced ferroptosis. To this aim, total mRNA was extracted from CHL-1, SK-Mel 5, A375, A2058 and C8161 cells and the mRNA levels of MITF, SOX9, SOX10, SMAD3, CTNNB1, AXL, EGFR, and ERBB3^43^ were evaluated by qRT-PCR. Our comparative analysis was performed using the most resistant CHL-1 cell line as reference (which expression was set to 1, in each analysis; Fig. 5a). Surprisingly, results indicate a differential and extremely heterogeneous expression of indicated markers in our cell lines, with a no clear ‘differentiation signature’ and/or correlation with ferroptosis susceptibility (Fig. 5b). We therefore performed a PCA (Principal Component Assay) and a Hierarchical Clustering analysis of our data set. Both analyses reported in Fig. 5c, d show that the mild (as shown in Fig. 5a) resistant SK-Mel 5 are close related to resistant A375, thus being part of the same group, while the most resistant CHL-1 are surprisingly close related to the sensitive A2058, thus being part of a second group. Moreover, the most sensitive C8161 is approximately equally distant from both previous groups, although more related to the A375/SK-Mel 5 than the CHL-1/A2058 group (Fig. 5c, d).

We can conclude that Graeber’s ‘differentiation signature’ and correlation with cell’s sensitivity to ferroptosis cannot be applied to our panel of metastasis-derived melanoma cells. This can potentially be explained, at least in part, by the heterogeneous source of our cells, such as lymph nodes for A2058 and SK-Mel 5, abdominal wall metastatic site for C8161, skin for A375, and pleural effusion metastatic site for CHL-1^44^.

However, during this screening we observed a differential basal expression of another gene implicated in the ferroptotic process, SLC7A11 (part of the X_C_^-^ system multiprotein complex; Fig. 5e) that correlates with our cell line sensitivity to ferroptosis execution (*R*^2^ = 0,7874; Fig. 5f). We also measured the basal expression of another gene previously proposed as ferroptosis sensitivity marker, ACSL4^45^, and confirmed that the expression of this factor is preferentially enhanced also in ferroptosis sensitive melanoma cells, compared to resistant ones (Suppl. S11). However, the correlation with cell death seems to be less accurate compared to SLC7A11 (*R*^2^ = 0,7874 vs *R*^2^ = 0.4962, referred to ACSL4).

Therefore, although future studies are required to better define the meaning of this correlation at molecular level, we can speculate SLC7A11 might represent a useful marker to predict metastatic melanoma cells susceptibility to ferroptosis execution, possibly coupled to ACSL4 gene expression analysis (Suppl S12).

### AKR1C1 ÷ 3 are expressed at low level in melanoma tumor in vivo

**Fig. 6:**
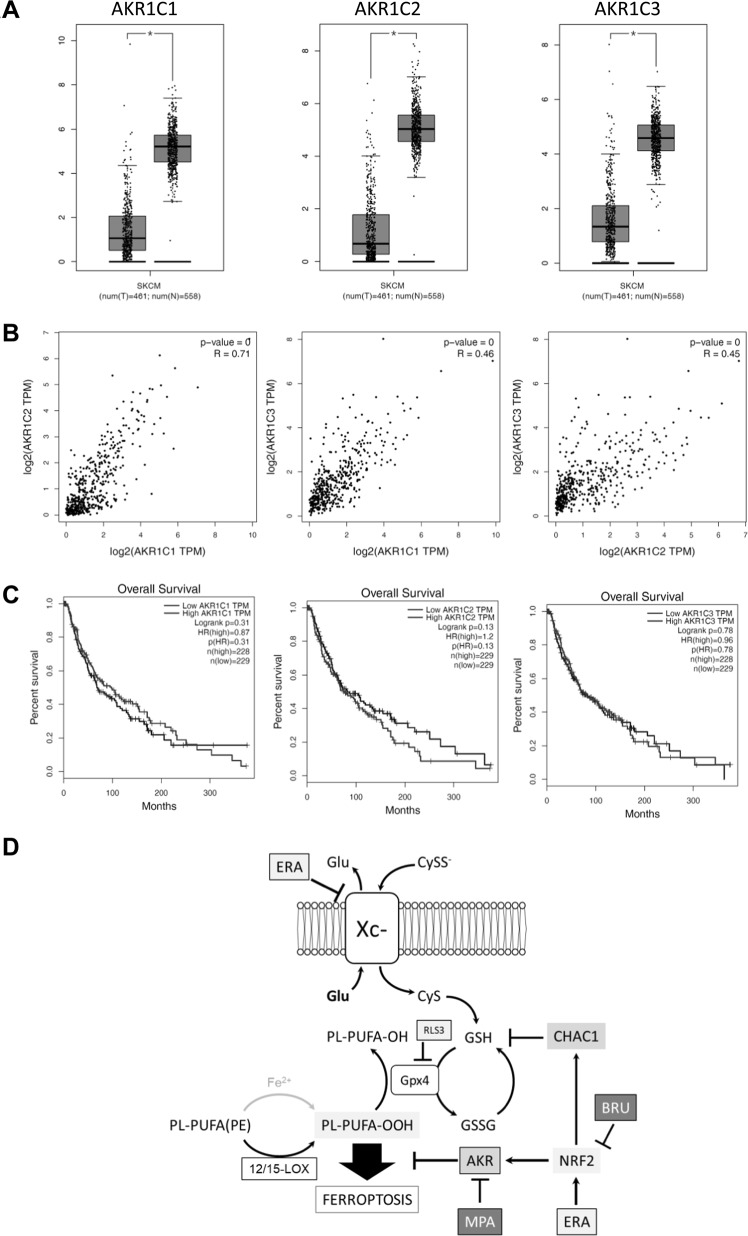
Gene expression profiling analysis. **a** A comparative expression analysis of AKR1C1, AKR1C2 and AKR1C3 was evaluated SKCM and normal tissue data sets. **b** A correlative analysis between AKR1C1 vs AKR1C2, AKR1C1 vs AKR1C3 and AKR1C2 vs AKR1C3 was performed in the SKCM data set. **c** The overall survival was evaluated in both high and low expressing AKR1C1, AKR1C2 or AKR1C3 patients. The total number of samples analyzed was indicated under each boxplot. **d** Graphic illustration showing the molecular mechanisms conferring resistance of metastatic melanoma cells to the execution of ferroptosis, and potential therapeutic interventions to counteract and/or resensitize cancer cells to ferroptosis.

The expression of AKR1C ÷ 3 was also evaluated in primary melanoma tumor samples compared to normal tissues, by using the GEPIA (Gene Expression Profiling Interactive Analysis) free online platform, available at http://gepia.cancer-pku.cn/index.html^46^. The analysis was carried out on 461 tumor and 558 normal tissues samples. Thus, the comparative analysis revealed that the three enzymes are expressed in both tumor and normal tissue but, interestingly, their level is statistically significant lower in tumor compared to normal tissue samples (Fig. 6a), while very low expression and not statistically significant difference are observed analyzing the expression of the unrelated AKR1C4 member (Suppl. S13A). Importantly, there is a clear correlation between the expression of each of the three AKRs and the other two members of the same family of enzymes (Fig. 6b; *R* = 0,71 in C1 vs C2; *R* = 0,46 in C1 vs C3; *R* = 0,45 in C2 vs C3), while there is no correlation between each of the three enzymes and the expression of the unrelated C4 (Suppl. S13B; *R* = 0,098 in C1 vs C4; *R* = 0,078 in C2 vs C4; *R* = 0,091 in C3 vs C4). Moreover, no change was observed, in any of the three AKRs, during the tumor stage progression (Suppl. S13C). Next, we also compared the overall survival from patients characterize by low vs high AKR1C1, AKR1C2, or AKR1C3. This analysis revealed that there is no significative difference linked to low or high AKR1C1 ÷ 3 expression (Fig. 6c). Due to our results indicating a NRF2-dependt AKR gene expression regulation under pro-ferroptotic drugs, we also evaluated the expression of this TF in the same data set reported above. This analysis indicates a slight reduced expression of NRF2 in tumor compared to normal tissue samples (Suppl. S13D) with no significant change during tumor stage progression (Suppl. S13E). Finally, our analysis also shows no link between overall patient’s survival rates and low/high NRF2 expression (Suppl. S13F). Finally, since 12/15-LOX are implicated in lipid peroxides generation under ferroptosis execution in melanoma cell lines, we also evaluated the expression of these factors in both tumor and normal tissue samples. Results indicate a reduced expression of both enzymes in tumor samples compared to normal tissues (Suppl. S13G) with no change during tumor stage progression (Suppl. S13H).

## Discussion

Human malignancies are currently one of the world’s worst problems that both clinicians and researchers face daily and although effective therapies have progressed thus sustaining patient’s survival in some of them, others are unfortunately still far from a definitive cure. Human skin melanoma is one of the latter, with an increasing incidence rate over years, high aggressiveness, still unfavorable clinical outcome, and refractory to any anti-tumor therapy developed till now. Therefore, there’s an urgent need of effective therapeutic strategies to treat this tumor. The deep dissection of molecular mechanisms responsible for melanomagenesis and tumor resistance to pro-death drugs will help to define new targets and more effective therapeutic strategies. Here we explored an alternative strategy to kill melanoma cells based on the induction of the unconventional cell death pathway named ferroptosis. Interestingly we found that this non-apoptotic signaling pathway is efficiently induced in all cells of our panel of commercially available melanoma cell lines, as evidenced by the fast, consistent and sustained upregulation of the early marker CHAC1. It has been previously reported a potential link existing between the apoptotic and ferroptotic cell death pathways relaying on the activation of ER stress, also possibly responsible for CHAC1 gene expression modulation under ferroptotic conditions^47^. However, we found that the UPR signaling pathway is not significantly induced upon ferroptosis induction in our panel of melanoma cell lines, thus indicating the existence of an ER stress-independent upregulation of CHAC1 under ferroptotic conditions. In fact, the inhibition of the PERK/ATF4/CHOP pathways, known to regulate CHAC1 expression^27,28^, did not result in gene expression abrogation of this marker upon ferroptosis induction. These results are of particular interest since dysregulated UPR is frequently involved in cancer cell development and resistance to therapy^48^, and has a pivotal role in oncogenic BRAF melanoma cells resistance to pro-apoptotic stimuli^13^.

Although competent in inducing the early stages of ferroptosis, most of our cell lines (5 out of 7) were unable to die though ferroptosis, independently from oncogenic BRAF expression and kinase activity inhibition (through short-term incubation with vemurafenib; 24 h), indicating a downstream block of the pathway. In fact, resistant cells showed low production of lipid peroxides, the key executioner of this cell death program, compared to sensitive cells, and only resistant cells were characterized by fast, consistent and sustained expression of AKRs (AKR1C1 ÷ 3), potentially responsible for lipid peroxides reduction and, thus, cell death inhibition. The inhibition of AKRs enzymatic activity or gene expression confirmed the latter hypothesis. Indeed, the AKRs pan inhibitor MPA or gene silencing (through shRNA) were able to restore the production of high levels of lipid peroxides resulting in cell death execution of all resistant cells. Therefore, we found new potential target to revert ferroptosis resistance, at least in melanoma cells. Further studies are, however, required to verify whether this resistance strategy is peculiar of melanoma cells or might represent a general cancer cell strategy to escape a pro-ferroptotic treatment. Interestingly, we also observed a heterogeneous extent of AKRs upregulation upon ferroptosis induction among resistant cells, with a ‘bona fide’ inverse correlation with their basal expression. The key role of AKRs in lipid peroxide detoxification and ferroptosis resistance was finally evidenced in sensitive cells in which the ectopic expression of one of the AKRs resulted in the conversion of sensitive into resistant cells and, importantly, the inhibition of its enzymatic activity completely reverted their phenotype. Interestingly, the analysis of AKRs expression performed by the Gene Expression Profiling Interactive Analysis (GEPIA) revealed that the three enzymes (AKR1C1 ÷ 3) are expressed at low level in tumor samples compared to normal tissue with no correlation between gene expression and patient’s survival rates. Further *in vivo* studies are therefore required to verify the enhanced expression of AKRs in ferroptotic resistant patients.

Although lipid peroxide generation was originally linked to intracellular iron accumulation, through the Fenton’s reaction^32,49^, it now generally accepted that these key ferroptotic executioner can also be generated by lipoxygenases. This is also the case of melanoma cells since the inhibition of 12/15-LOX but not 5-LOX resulted in both complete abrogation of lipid peroxides production and cell death execution under ferroptotic treatment in both sensitive and MPA-treated resistant cells.

Finally, our current study indicates that NRF2 is actively involved in melanoma cell resistance to ferroptotic cell death since its expression, together with its downstream target HO1, increased at both mRNA and protein levels in resistant cells, upon treatment. Importantly, the pharmacologic inhibition of NRF2 activity inhibited the ferroptosis-induced upregulation of AKRs. Moreover, the inhibition of NRF2 activity also resulted in complete abrogation of CHAC1 early upregulation upon ferroptosis induction, thus delineating a new rote though which CHAC1 expression is modulated during ferroptosis induction/execution (Fig. 6d).

During the preparation of this manuscript, Graeber and colleagues published data showing a correlation between primary tumor-derived melanoma cell’s differentiation and ferroptosis resistance, identifying a panel of genes which expression well recapitulated the differentiation status of these cells^43^. However, this ‘differentiation signature’ seems not to be applicable to metastasis-derived melanoma cells possibly because the latter are not derived (most of them) by primary tumors but from secondary metastatic sites. Consequently, since the metastatic phenotype is associated with an epithelial–mesenchymal transition (EMT), this implies a cells reprogramming thus resulting in a new repertoire of expressed genes, to support specific adhesive, invasive, and migratory properties^50^.

However, although Graeber’s ‘differentiation signature’ failed in defying the differentiation status of our cells and the consequent relation with ferroptosis resistance, we found a positive correlation between the basal expression of SLC7A11 (a member of the System X_C_^-^) and cell’s resistance to ferroptosis execution. Therefore, although further studies are required, and an extended panel of metastatic melanoma cell lines should be screened to verify this relation, it is possible to speculate that this factor might represent a new potential marker to predict metastatic melanoma sensitivity to ferroptotic cell death, possibly coupled to other potential markers such as ACSL4. Interestingly, very recently Zhang and colleagues showed a link between the tumor suppressor BRCA1-associated protein 1 (BAP1) mutational status and SLC7A11 expression in uveal melanoma^51^. However, data on BAP1 mutational status in skin melanoma are still missing. Our work thus motivates further studies to elucidate the potential link between BAP1 and SLC7A11 in human skin melanoma.

To the best of our knowledge ferroptosis resistance has not previously been associated with AKRs upregulation/activation. Our study thus identifies a new potential therapeutic strategy to efficiently kill melanoma cells based on pro-ferroptosis drugs coupled to AKR1C1 ÷ 3 inhibitors (Fig. 6d).

## Methods and materials

### Cell culture and treatments

Human melanoma cell lines used were: MeWo, A2058, SK-Mel 5, SK-Mel 24, C8161, CHL-1, and A375. Cell identity was confirmed by short tandem repeat analysis (STR) and the DSMZ Online STR Analysis^52^, and Mycoplasma testing was routinely performed each month by using the Venor®GeM Classic (Minerva-BiolAbs; Berlin, GE).

Cell lines (BRAF^WT^: CHL-1, SK-Mel 24 and MeWo; BRAF^V600E^: A375, A2058 and SK Mel-5; BRAF^G464E^: C8161) were cultured in DMEM (Sigma-Aldrich; Milan, IT), supplemented with 10% foetal bovine serum (Sigma-Aldrich), 2mM L-glutamine (Sigma-Aldrich), 1% penicillin/streptomycin solution (Sigma-Aldrich) at 37 °C under 5% CO_2_. All reagents were purchased from Sigma-Aldrich if not differently indicated. Cells were treated with Thapsigargin 10 μg/ml, Erastin 10 μM, Brusatol 50 nM (Cayman Chemicals; Ann Arbor, MI, US); Medroxyprogesterone 10 μM, Baicalein 20 μM, MG132 10μM, H_2_O_2_ 500 μM; Deferoxamine 100μM (Cayman Chemicals); Zileuton 10 μM (Cayman Chemicals); Ferrostatin-1 10 μM; Vemurafenib 10 μM; 2-Mercaptoethanol 100μM; RLS3 2μM (Sigma-Aldrich).

### Lentiviral generation and infection

Co-transfection of lentiviral vectors (shRNA-pLKOs AKR1C1, shRNA-pLKOs AKR1C2, shRNA-pLKO AKR1C3; Sigma-Aldrich) (10 μg), vesicular stomatitis virus G protein expression plasmid (2,5 μg) and psPAX2 plasmid (carrying gag, pol and rev genes) was performed using 293T packaging cell line, by a calcium phosphate protocol. Supernatants with lentiviral particles were harvested 48 h later and supplemented with 4 μg/ml of polybrene. These supernatants were used to infect target cells^30^.

### RNA interference

hATF4 and non-targeting scramble (siCTRL, used as negative control) siRNA oligoribonucleotides were obtained from Invitrogen (Carlsbad, CA, US). Silencing was performed as previously reported. Briefly, 25 × 10^4^ cells/well were seeded in six-well plates and transfected with siRNA (100 pmol) by means of RNAi Max (Invitrogen) as recommended by the supplier. 24 h later, cells were trypsinized, plated at 30 × 10^4^ cells/well in six-well plates and treated with the indicated agents. Quantitative RT-PCR (qRT-PCR) analysis was used to assess RNA downregulation after 48 h from transfection, as described below.

### Cell transfection

AKR1C3 encoding vector (RC200210–AKR1C3 (NM_003739) Human Tagged ORF Clone) was purchased from OriGene (Rockville, MD, US). A total of 25 × 10^4^ cells/well were transfected with 1 µg of total DNA in six-well plates by using Lipofectamine LTX (Invitrogen) for 8 h, as recommended by the supplier. At 24 h after transfection, cells were trypsinized, plated at 30 × 10^4^ cells/well in six-well plates and treated as indicated.

### Western blotting

Protein extraction was performed by using Cell Lytic buffer (Sigma-Aldrich) supplemented with a protease inhibitors cocktail (Sigma-Aldrich) plus phosphatases inhibitors (Na3VO4 1 mM; NaF 10 mM) and resolved by electrophoresis through NuPAGE Bis-Tris gel (Invitrogen) and electroblotted onto nitrocellulose (Protran, Sigma-Aldrich) membrane. Blots were incubated with indicated primary antibodies in 5% non-fat dry milk in PBS plus 0.1% Tween20 overnight at 4 °C. Primary antibodies were: anti-PERK (1:500; Cell Signaling; Danvers, MA, US), anti-ERK1/2 (1:500; Cell Signaling), anti-ERK1/2 (1:500; Cell Signaling), anti-Nrf2 (1:500; Genetex; Alton Pkwy Irvine, CA, US), anti-Herp (1:500; Sigma-Aldrich), anti-AKR1C1 (1:500; NOVUS), anti-AKR1C2 (1:500, Merck), anti-AKR1C3 (1:500; Cell Signaling), anti-Tubulin-α (1:5000; Santa Cruz Biotechnology; Santa Cruz, CA, US). Detection was achieved using horseradish peroxidase-conjugate secondary antibody (1:5000; Jackson ImmunoResearch; Cambridge, UK) and visualized with ECL plus (Amersham Biosciences; Amersham, UK). Images were acquired by using a ChemiDoc™ Touch Imaging System (Bio-Rad; Berkeley, CA, US) and analyzed by Image Lab software (Bio-Rad).

### Quantitative RT-polymerase chain reaction

Trizol reagent (Invitrogen) was used to extract total RNA as indicated by the supplier, and the AMV Reverse Transcriptase kit (Promega; Madison, WI, US) was used to produce cDNA following the manufacturer’s recommendations. Quantitative PCR reactions were performed by using the Rotor-Gene 6000 (Corbett Research Ltd; Cambridge, UK) thermocycler. Supplementary Table S1 shows the primer pair sequences for all amplicons, designed by using the online IDT PrimerQuest Tool software (IDT; https://eu.idtdna.com/Primerquest/Home/Index). L34 mRNA level was used as an internal control and results were expressed as previously described.

### Cell viability

Fluorescein diacetate (FDA)/7AAD staining was used to identify and measure the percentage of live/dead cells. Briefly, cells were incubated (10 min) with PBS containing FDA (7 pg/ml) and 7AAD (50 ng/ml) and 10’000 events were acquired by flow cytometry. The percentage of FDA positive and 7AAD negative cells was measured and indicated as ‘Cell Viability (%)’.

### Lipid peroxides evaluation

Briefly, 1.5 × 10^5^ cells were treatment as indicated and cells harvested at indicate time points. Then, cells were pelleted, washed by PBS, resuspended in BODIPY C11 (2 μM in PBS; Invitrogen), incubated at 37 °C for 15 min in the dark, and 10.000 events were acquired by using a FACS Calibur cytometer (Becton-Dickinson; Franklin Lakes, NJ, US). Data analysis was performed using the Flowing Software.

### PCA and hierarchical cluster analysis

The original expression data for the panel composed by the eight genes across the five cell lines reported in Supplementary Table S2 were subject to two different analysis: a PCA (Principal Component Analysis) and a hierarchical clustering (Euclidean clustering distance, complete linkage). Data were log2 transformed and scaled with respect to the gene median value. A pseudo-count of 0.01 was added to all samples. All the computations were performed in the R statistical environment (https://www.r-project.org/).

### Gene expression profiling interactive analysis (GEPIA)

The analysis was performed by using the free online web platform at http://gepia.cancer-pku.cn/index.html, which details are available at http://gepia.cancer-pku.cn/help.html and^46^. Briefly, the expression of each indicated gene was evaluated in the skin melanoma, SKCM, data set matched with ‘TCGA normal and GTEx data’ set (normal tissue), with a *|Log*_*2*_*FC| Cutoff* of 1, a *p-value Cutoff* of 0.01, and results showed using a *log2(TPM* *+* *1)* log scale. The overall survival curves were generated by using the following parameters: Group Cutoff = Median, with Cutoff-High (%) = 50 and Cutoff-Low (%) = 50; Hazards Ratio (HR) = Yes. Gene correlation analysis was performed in the SKCM data set by using the Pearson Correlation Coefficient, with a non-log scale for calculation and a log-scale axis for visualization.

### Statistical analysis

All experiments were performed at least triplicate. Data showed in this paper are representative of 3 independent experiments carried out in triplicate; western blotting images are from a representative experiment carried out in triplicate. Statistical analysis was performed using GraphPad Prism 6 and Student’s *t* test was used to determine statistical significance. A *p*-value of equal to or less than 0.05 was considered significant.

## Supplementary information

Supporting Information Methods

Supplementary Table S1

Supplementary Table S2

Supplementary Figures S1-13

## Data Availability

The data supporting the conclusion of this research has been included in this published article and its additional files.

## References

[1] Thompson JF, Scolyer RA, Kefford RF (2005). Cutaneous melanoma. Lancet (London, England).

[2] Corazzari, M., Fimia, G. M., Lovat, P. & Piacentini, M. Why is autophagy important for melanoma? Molecular mechanisms and therapeutic implications. *Semin. Cancer Biol*. **23**, 337–343 (2013). 10.1016/j.semcancer.2013.07.001 23856558

[3] Fecher LA, Cummings SD, Keefe MJ, Alani RM (2007). Toward a molecular classification of melanoma. J. Clin. Oncol..

[4] Arkenau H-T, Kefford R, Long GV (2011). Targeting BRAF for patients with melanoma. Br. J. Cancer.

[5] Soengas MS (2001). Inactivation of the apoptosis effector Apaf-1 in malignant melanoma. Nature.

[6] Reddy BY, Miller DM, Tsao H (2017). Somatic driver mutations in melanoma. Cancer.

[7] Flaherty KT (2012). Combined BRAF and MEK Inhibition in Melanoma with BRAF V600 Mutations. N. Engl. J. Med..

[8] Cantwell-Dorris ER, O’Leary JJ, Sheils OM (2011). BRAFV600E: implications for carcinogenesis and molecular therapy. Mol. Cancer Ther..

[9] Aguissa-Touré A-H, Li G (2012). Genetic alterations of PTEN in human melanoma. Cell. Mol. Life Sci..

[10] Dhomen N, Marais R (2009). BRAF signaling and targeted therapies in melanoma. Hematol. Oncol. Clin. North Am..

[11] Lazova R, Klump V, Pawelek J (2010). Autophagy in cutaneous malignant melanoma. J. Cutan. Pathol..

[12] Ma X-H (2011). Measurements of tumor cell autophagy predict invasiveness, resistance to chemotherapy, and survival in melanoma. Clin. Cancer Res..

[13] Corazzari M (2015). Oncogenic BRAF induces chronic ER stress condition resulting in increased basal autophagy and apoptotic resistance of cutaneous melanoma. Cell Death Differ..

[14] Armstrong J. L., Corazzari M., Martin S., Pagliarini V., Falasca L., Hill D. S., Ellis N., Al Sabah S., Redfern C. P. F., Fimia G. M., Piacentini M., Lovat P. E. (2011). Oncogenic B-RAF Signaling in Melanoma Impairs the Therapeutic Advantage of Autophagy Inhibition. Clinical Cancer Research.

[15] Giglio P, Fimia GM, Lovat PE, Piacentini M, Corazzari M (2015). Fateful music from a talented orchestra with a wicked conductor: connection between oncogenic BRAF, ER stress, and autophagy in human melanoma. Mol. Cell. Oncol..

[16] Gowrishankar K (2012). Acquired resistance to BRAF inhibition can confer cross-resistance to combined BRAF/MEK inhibition. J. Invest. Dermatol..

[17] Klionsky, D. J. et al. Guidelines for the use and interpretation of assays for monitoring autophagy (3rd edition). *Autophagy* **12**, 1–222 (2016). 10.1080/15548627.2015.1100356 PMC4835977 26799652

[18] Fedorenko IV, Wargo JA, Flaherty KT, Messina JL, Smalley KSM (2015). BRAF inhibition generates a host–tumor niche that mediates therapeutic escape. J. Invest. Dermatol..

[19] Topalian SL (2012). Safety, activity, and immune correlates of anti–PD-1 antibody in cancer. N. Engl. J. Med..

[20] Hodi FS (2010). Improved survival with ipilimumab in patients with metastatic melanoma. N. Engl. J. Med..

[21] Schachter J (2017). Pembrolizumab versus ipilimumab for advanced melanoma: final overall survival results of a multicentre, randomised, open-label phase 3 study (KEYNOTE-006). Lancet.

[22] Hoos A (2016). Development of immuno-oncology drugs—from CTLA4 to PD1 to the next generations. Nat. Rev. Drug Discov..

[23] Hugo W (2017). Genomic and transcriptomic features of response to anti-PD-1 therapy in metastatic melanoma. Cell.

[24] Dixon SJ (2012). Ferroptosis: an iron-dependent form of nonapoptotic cell death. Cell.

[25] Dixon SJ (2014). Pharmacological inhibition of cystine–glutamate exchange induces endoplasmic reticulum stress and ferroptosis. Elife.

[26] Gaschler MM, Stockwell BR (2017). Lipid peroxidation in cell death. Biochem. Biophys. Res. Commun..

[27] Mungrue IN, Pagnon J, Kohannim O, Gargalovic PS, Lusis AJ (2009). CHAC1/MGC4504 is a novel proapoptotic component of the unfolded protein response, downstream of the ATF4-ATF3-CHOP cascade. J. Immunol..

[28] Crawford RR (2015). Human CHAC1 protein degrades glutathione, and mRNA Induction is regulated by the transcription factors ATF4 and ATF3 and a bipartite ATF/CRE regulatory element. J. Biol. Chem..

[29] Yamamoto K, Yoshida H, Kokame K, Kaufman RJ, Mori K (2004). Differential contributions of ATF6 and XBP1 to the activation of endoplasmic reticulum stress-responsive cis-acting elements ERSE, UPRE and ERSE-II. J. Biochem..

[30] Giglio, P. et al. PKR and GCN2 stress kinases promote an ER stress-independent eIF2α phosphorylation responsible for calreticulin exposure in melanoma cells. *Oncoimmunology* (2018). 10.1080/2162402X.2018.1466765 10.1080/2162402X.2018.1466765 PMC6136861 30221067

[31] Penning TM (2015). The aldo-keto reductases (AKRs): overview. Chem. Biol. Interact..

[32] Latunde-Dada GO (2017). Ferroptosis: role of lipid peroxidation, iron and ferritinophagy. Biochim. Biophys. Acta - Gen. Subj..

[33] Barski OA, Tipparaju SM, Bhatnagar A (2008). The aldo-keto reductase superfamily and its role in drug metabolism and detoxification. Drug Metab. Rev..

[34] Yang WS, Stockwell BR (2016). Ferroptosis: death by lipid peroxidation. Trends Cell Biol..

[35] Hao S (2018). Metabolic networks in ferroptosis (Review). Oncol. Lett..

[36] Shintoku R (2017). Lipoxygenase-mediated generation of lipid peroxides enhances ferroptosis induced by erastin and RSL3. Cancer Sci..

[37] Xu J (2013). Inhibition of 12/15-lipoxygenase by baicalein induces microglia PPARβ/δ: a potential therapeutic role for CNS autoimmune disease. Cell Death Dis..

[38] Nguyen T, Nioi P, Pickett CB (2009). The Nrf2-antioxidant response element signaling pathway and its activation by oxidative stress. J. Biol. Chem..

[39] Penning TM (2017). Aldo-keto reductase regulation by the Nrf2 system: implications for stress response, chemotherapy drug resistance, and carcinogenesis. Chem. Res. Toxicol..

[40] Shibata T (2008). Genetic alteration of Keap1 confers constitutive Nrf2 activation and resistance to chemotherapy in gallbladder cancer. Gastroenterology.

[41] Miura S (2014). A somatic mutation of the KEAP1 gene in malignant melanoma is involved in aberrant NRF2 activation and an increase in intrinsic drug resistance. J. Invest. Dermatol..

[42] Ren D (2011). Brusatol enhances the efficacy of chemotherapy by inhibiting the Nrf2-mediated defense mechanism. Proc. Natl Acad. Sci. U. S. A..

[43] Tsoi J (2018). Multi-stage differentiation defines melanoma subtypes with differential vulnerability to drug-induced iron-dependent oxidative stress. Cancer Cell.

[44] Su DM (2009). Two types of human malignant melanoma cell lines revealed by expression patterns of mitochondrial and survival-apoptosis genes: implications for malignant melanoma therapy. Mol. Cancer Ther..

[45] Doll S (2017). ACSL4 dictates ferroptosis sensitivity by shaping cellular lipid composition. Nat. Chem. Biol..

[46] Tang Z (2017). GEPIA: a web server for cancer and normal gene expression profiling and interactive analyses. Nucleic Acids Res..

[47] Hong SH (2017). Molecular crosstalk between ferroptosis and apoptosis: emerging role of ER stress-induced p53-independent PUMA expression. Oncotarget.

[48] Corazzari, M., Gagliardi, M., Fimia, G. M. & Piacentini, M. Endoplasmic reticulum stress, unfolded protein response, and cancer cell fate. *Front. Oncol*. **7**, 78 (2017). 10.3389/fonc.2017.00078 PMC5405076 28491820

[49] Doll S, Conrad M (2017). Iron and ferroptosis: a still ill-defined liaison. IUBMB Life.

[50] Alonso SR (2007). A high-throughput study in melanoma identifies epithelial-mesenchymal transition as a major determinant of metastasis. Cancer Res..

[51] Zhang Y (2018). BAP1 links metabolic regulation of ferroptosis to tumour suppression. Nat. Cell Biol..

[52] Dirks WG (2010). Cell line cross-contamination initiative: an interactive reference database of STR profiles covering common cancer cell lines. Int. J. Cancer.

