# Supplementary material for: Aldo-keto reductases protect metastatic melanoma from ER stress-independent ferroptosis
Source: Cell Death Dis. 2019 Nov 28;10(12):902. doi: 10.1038/s41419-019-2143-7 (PMC6883066; doi:10.1038/s41419-019-2143-7)
Supplement: Supplementary file 1 — Supporting Information Methods [file 41419_2019_2143_MOESM1_ESM.pdf]

Aldo-keto reductases protect metastatic melanoma from ER stressindependent ferroptosis

SUPPORTING INFORMATION METHODS

Cell culture and treatments

**A375**	**RRID:CVCL_0132**	$BRAF_{V600E}$
**CHL-1**	**RRID:CVCL_1122**	$BRAF^{WT}$
**A2058**	**RRID:CVCL_1059**	$BRAF_{V600E}$
**C8161**	**RRID:CVCL_6813**	$BRAF_{G464E}$
**MeWo**	**RRID:CVCL_0445**	$BRAF^{WT}$
**SK-Mel-5**	**RRID:CVCL_0527**	$BRAF_{V600E}$
**SK-Mel24**	**RRID:CVCL_0599**	$BRAF^{WT}$

A2058, C8161, MeWo, SK-M2l-5 and SK-Mel-24 were purchased by The Biological Bank Core Facility, IRCCS Azienda Ospedaliera Universitaria San Martino – IST Istituto Nazionale per la Ricerca sul Cancro, Genova,IT; A375 and CHL-1 were kindly provided by Prof. P. Lovat, University of Newcastle, Newcastle Upon Tyne, UK).

Cell lines were cultured in DMEM (Sigma-Aldrich), supplemented with 10% foetal bovine serum (Sigma-Aldrich), 2mM L-glutamine (Sigma-Aldrich), 1% penicillin/streptomycin solution (SigmaAldrich) at 37 °C under 5% CO_2. All reagents were purchased from Sigma-Aldrich if not differently indicated. Cells were treated with Thapsigargin 10μg/ml, Erastin 10μM, 10μM, Brusatol 50nM (Cayman Chemicals); Medroxyprogesterone 10μM, Baicalein 20μM, MG132 10μM, H_2O_2 500μM; Deferoxamine 100μM (Cayman Chemicals); Zileuton 10μM (Cayman Chemicals); Ferrostatin-1 10 μM; Vemurafenib 10 μM; 2-Mercaptoethanol 100μM; RLS3 2μM.

Lentiviral generation and infection

Co-transfection of lentiviral vectors (shRNA-pLKOs AKR1C1, shRNA-pLKOs AKR1C2, shRNApLKO AKR1C3; Sigma-Aldrich) (10 µg), vesicular stomatitis virus G protein expression plasmid (2,5 µg) and psPAX2 plasmid (carrying gag, pol and rev genes) was performed using 293T packaging cell line, by a calcium phosphate protocol. Supernatants with lentiviral particles were harvested 48h later and supplemented with 4 µg/ml of polybrene. These supernatants were used to infect target cells [31].

RNA interference

hATF4 and non-targeting scramble (siCTRL, used as negative control) siRNA oligoribonucleotides were obtained from Invitrogen. Silencing was performed as previously reported. Briefly, 25 x 10^4 cells/well were seeded in six-well plates and transfected with siRNA (100 pmol) by means of RNAi Max (Invitrogen) as recommended by the supplier. 24h later, cells were trypsinized, plated at 30 x 10^4 cells/well in six-well plates and treated with the indicated agents. Quantitative RT-PCR (qRTPCR) analysis was used to assess RNA down-regulation after 48h from transfection, as described below.

Western blotting

Protein extraction was performed by using Cell Lytic buffer (Sigma-Aldrich) supplemented with a protease inhibitors cocktail (Sigma-Aldrich) plus phosphatases inhibitors (Na3VO4 1mM; NaF 10mM) and resolved by electrophoresis through NuPAGE Bis-Tris gel (Invitrogen) and electroblotted onto nitrocellulose (Protran, Sigma-Aldrich) membrane. Blots were incubated with indicated primary antibodies in 5% non-fat dry milk in PBS plus 0.1% Tween20 overnight at 4° C. Primary antibodies were: anti-PERK (1:500; Cell Signaling), anti-ERK1/2 (1:500; Cell Signaling), anti-Nrf2 (1:500; Genetex), anti-Herp (1:500; Sigma-Aldrich), anti-ubulin-α (1:5000; Santa Cruz Biotechnology). Detection was achieved using horseradish peroxidase-conjugate secondary antibody (1:5000; Jackson ImmunoResearch) and visualized with ECL plus (Amersham Biosciences). Images were

acquired by using a ChemiDoc™ Touch Imaging System (Bio Rad) and analyzed by Image Lab software (Bio Rad).

Gene Expression Profiling Interactive Analysis (GEPIA)

The analysis was performed by using the free online web platform at http://gepia.cancerpku.cn/index.html, which details are available at http://gepia.cancer-pku.cn/help.html and [47]. Briefly, the expression of each indicated gene was evaluated in the skin melanoma, SKCM, data set matched with 'TCGA normal and GTEx data' set (normal tissue), with a $|Log_2FC|$ *Cutoff* of 1, a *pvalue Cutoff* of 0.01, and results showed using a *log2(TPM + 1)* log-scale. The overall survival curves were generated by using the following parameters: Group Cutoff = Median, with Cutoff-High (%) = 50 and Cutoff-Low (%) = 50; Hazards Ratio (HR) = Yes. Gene correlation analysis was performed in the SKCM data set by using the Pearson Correlation Coefficient, with a non-log scale for calculation and a log-scale axis for visualization.
