# Supplementary material for: Aldo-keto reductases protect metastatic melanoma from ER stress-independent ferroptosis
Source: Cell Death Dis. 2019 Nov 28;10(12):902. doi: 10.1038/s41419-019-2143-7 (PMC6883066; doi:10.1038/s41419-019-2143-7)
Supplement: Supplementary file 2 — Supplementary Table S1 [file 41419_2019_2143_MOESM2_ESM.pdf]

Table S1. qRT-PCR primers (5'→3')

L34 forward	GTCCCGAACCCCTGGTAATAGA
L34 reverse	GGCCCTGCTGACATGTTTCTT
CHAC1 forward	CTCAAGCGCTGTGGATTT
CHAC1 reverse	TGTCTCCCTGCCAGAAA
Xbp-I (spliced) forward	GAATGAAGTGAGGCCAGTG
Xbp-I (spliced) reverse	GAGTCAATACCGCCAGAATC
ATF4 forward	GTGGCCAAGCACTTCAAACC
ATF4 reverse	CCCGGAGAAGGCATCCTC
CHOP forward	AGAACCAGGAAACGGAAAC
CHOP reverse	GCTTGAGCCGTTCATTCT
ATF6 forward	TATCAGTTTACAACCTGCACCCACTA
ATF6 reverse	GCAAGGACTGGCTGAGCAGA
AKR1C1 forward	GCCGTGGAGAAGTGTAAAG
AKR1C1 reverse	CAGACAGGCTTGTACTTGAG
AKR1C2 forward	GGGTTCCACCATATTGATTCT
AKR1C2 reverse	CACTGCCATCTGCAATCT
AKR1C3 forward	CAGAGGTTCCGAGAAGTAAAG
AKR1C3 reverse	CCAACCTGCTCCTCATTATT
Nrf2 forward	CAACCCTTGTCACCATCTC
Nrf2 reverse	TTCCGATGACCAGGACTTA
HO1 forward	CCTCCCTGTACCACATCTAT
HO1 reverse	AGCTCTTCTGGGAAGTAGAC
MITF forward	GTCACTGATCCACTCCTTTC
MITF reverse	CGTCTCTTCCATGCTCATAC
SOX9 forward	CTACTCCACCTTCACCTACA
SOX9 reverse	GTGTGTAGACGGGTTGTTC

SOX10 forward	GCCCTCAGGACCCTATTAT
SOX10 reverse	AGAGATGGCCGTGTAGAG
SMAD3 forward	GTGTGAGTTCGCCTTCAATA
SMAD3 reverse	ACCAACACAGGAGGTAGAA
CTNNB1 forward	CTCAGATGGTGTCTGCTATTG
CTNNB1 reverse	ACGATGATGGGAAAGGTTATG
AXL forward	GTGGAGAACAGCGAGATTT
AXL reverse	GCGACATCAAGGCATACA
EGFR forward	GGTGGTCCTTGGGAATTTG
EGFR reverse	GAGGGCAATGAGGACATAAC
ERBB3 forward	GGGAAATACACACACCAGAG
ERBB3 reverse	GTCTGGTACTTCAGCCAATC
