# Supplementary material for: Aldo-keto reductases protect metastatic melanoma from ER stress-independent ferroptosis
Source: Cell Death Dis. 2019 Nov 28;10(12):902. doi: 10.1038/s41419-019-2143-7 (PMC6883066; doi:10.1038/s41419-019-2143-7)
Supplement: Supplementary file 3 — Supplementary Table S2 [file 41419_2019_2143_MOESM3_ESM.pdf]

Table S2. Median gene expression of the "differentiation markers"

		CELL LINES				
		CHL-1	A375	G361	A2058	C8161
GENES	MITF	1,01	9,79	56,30	3,75	0,05
	SOX9	0,83	8488,00	181,04	1,45	526,84
	SOX10	1,34	0,59	0,47	1,82	0,00
	SMAD3	1,13	41,23	18,84	13,89	17,45
	CTNNB1	1,04	0,59	2,32	1,34	0,24
	AXL	1,03	0,32	0,00	4,49	6,75
	EGFR	1,03	56,70	229,05	0,25	1067,59
	ERBB3	1,01	0,14	0,36	0,01	0,35
