# Supplementary material for: Aldo-keto reductases protect metastatic melanoma from ER stress-independent ferroptosis
Source: Cell Death Dis. 2019 Nov 28;10(12):902. doi: 10.1038/s41419-019-2143-7 (PMC6883066; doi:10.1038/s41419-019-2143-7)
Supplement: Supplementary file 4 — Supplementary Figures S1-13 [file 41419_2019_2143_MOESM4_ESM.pdf]

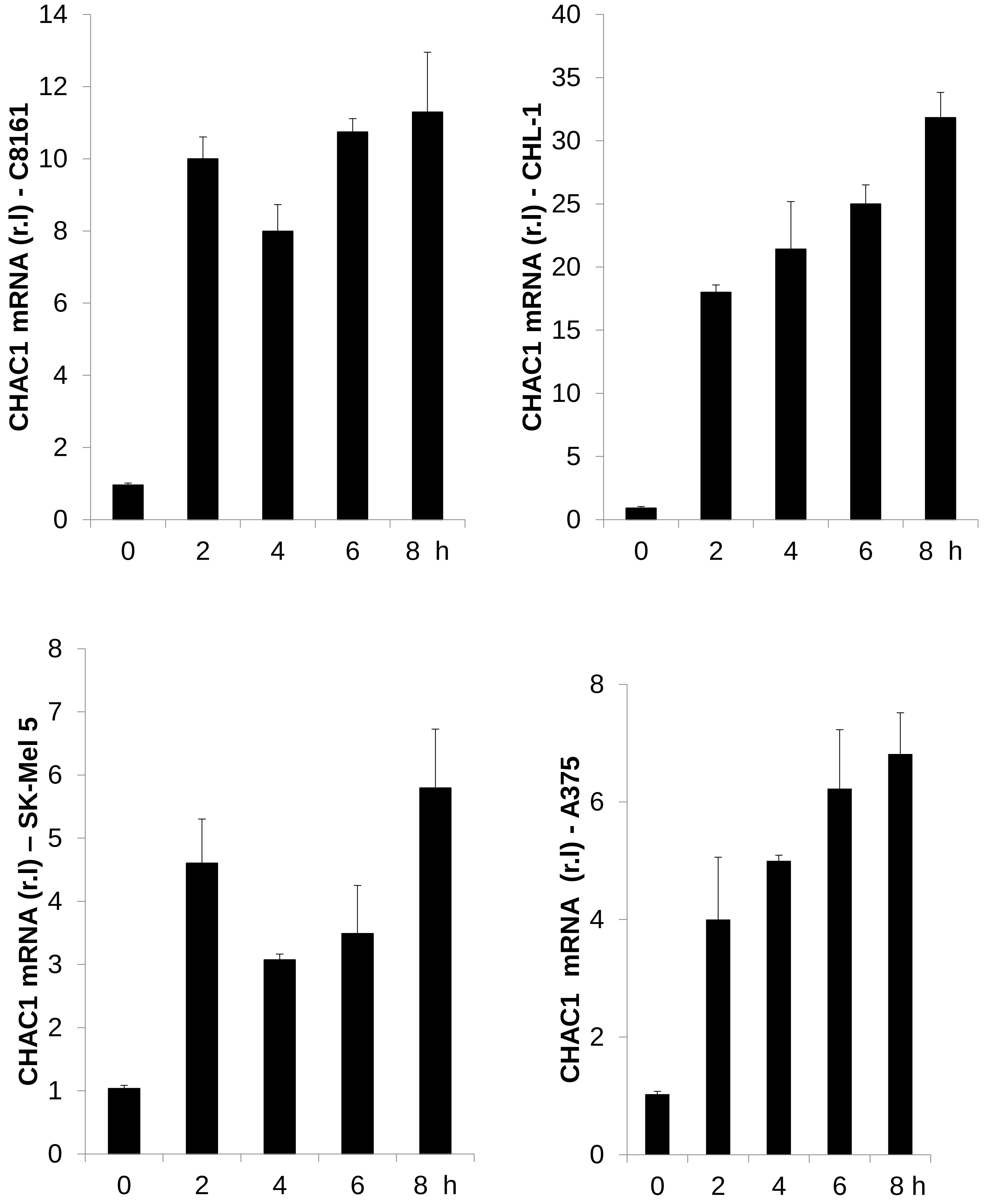

Supplementary Figure S1. Sustained enhanced expression of CHAC1 under ERA exposure in melanoma cells. C8161, CHL-1, SK-Mel 5 and A375 cells were exposed to ERA (10 μM) and CHAC1 expression was evaluated at indicated time points by qRT-PCR. (Histograms are representative of mean ± s.d.; n = 3).

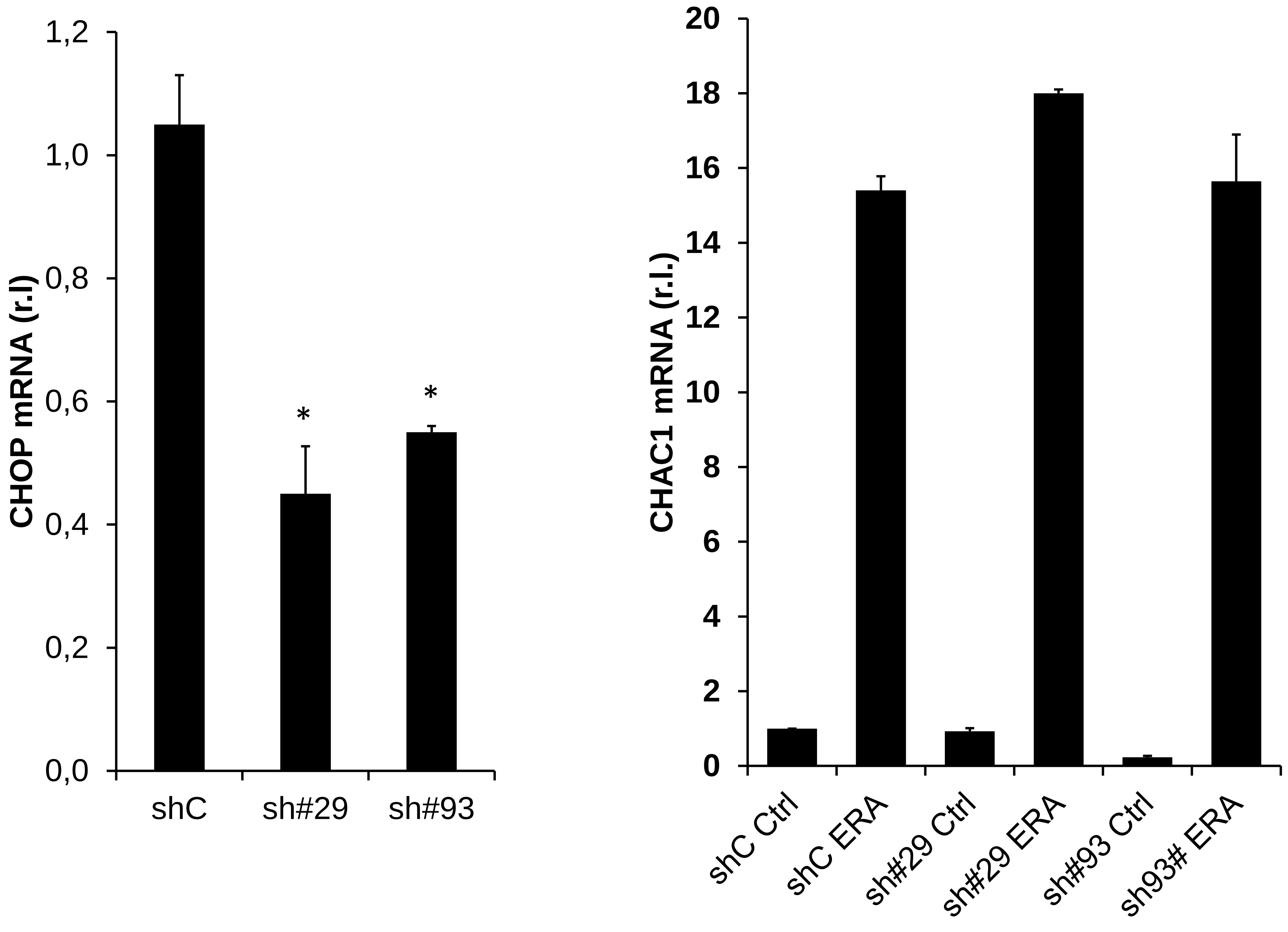

Supplementary Figure S2. CHOP/Gadd153 does not regulate CHAC1 gene expression under erastin treatment in melanoma cells. CHL-1 were infected with lentiviral particles carrying specific shRNA to target CHOP (sh#29 and sh#93) or a non specific scrambled sequence (shC). The expression of CHOP was evaluated 2 days post-infection by qRT-PCR (left panel), cells were then exposed to ERA for 2h and CHAC1 expression was evaluated by qRT-PCR (right panel). (Histograms are representative of mean ± s.d.; n = 3).

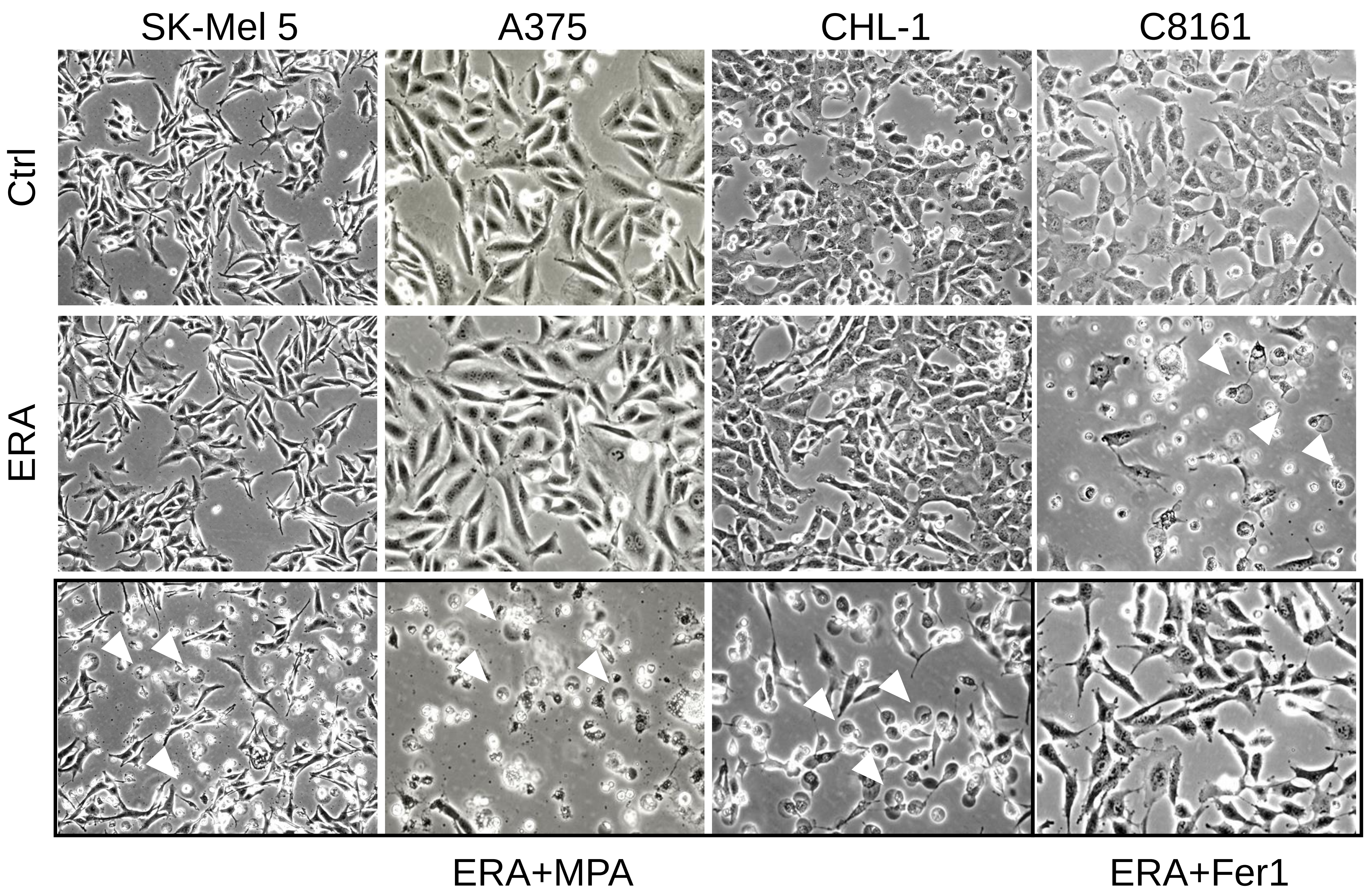

Supplementary Figure S3. Susceptibility of melanoma cells to ferroptotic cell death. Melanoma cell lines were exposed 24h to ERA (10 μM), MPA (10 μM), ERA+MPA or ERA+Fer1 (10 μM) as indicated and cell morphology was evaluated by light field microscopy. The appearance of membrane blebs were evidenced by white arrowheads. Images are representative of three independent experiments; magnification = 20x).

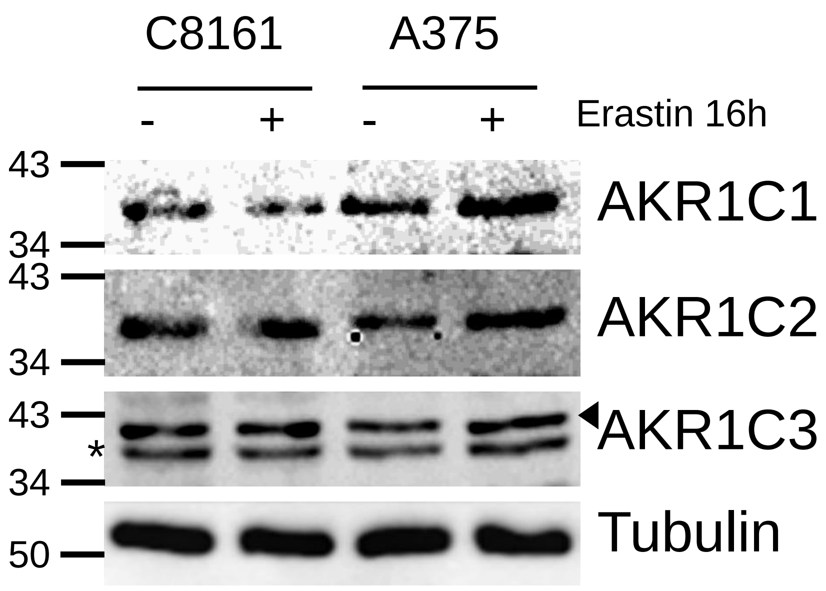

Supplementary Figure S4. AKR1Cs expression under ERA exposure. C8161 and A375 cells were incubated 16h with ERA (10 μM) or vehicle (DMSO) and 30 μg of total proteins were separated by SDS-gel electrophoresis. The expression levels of AKR1C1, AKR1C2 and AKR1C3 were evaluated by using specific antibodies. Tubulin was used as loading control. Images are representative of three independent experiments; * = non specific bands.

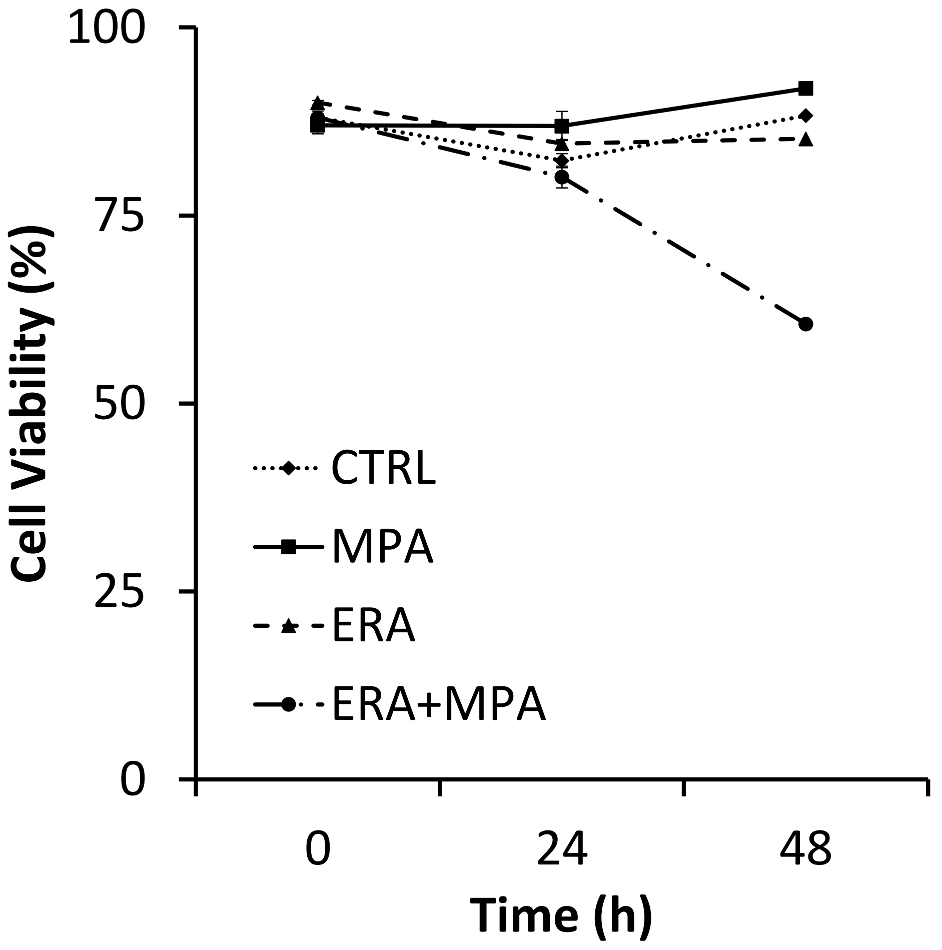

Supplementary Figure S5. AKRs inhibition enhances ferroptosis execution. SK-Mel 24 cells were treated or untreated with ERA, MPA alone or in combination (ERA+MPA) and cell viability was evaluated at 24 and 48h post-treatment, by flow cytometry. (Histograms are representative of mean ± s.d.; n = 3).

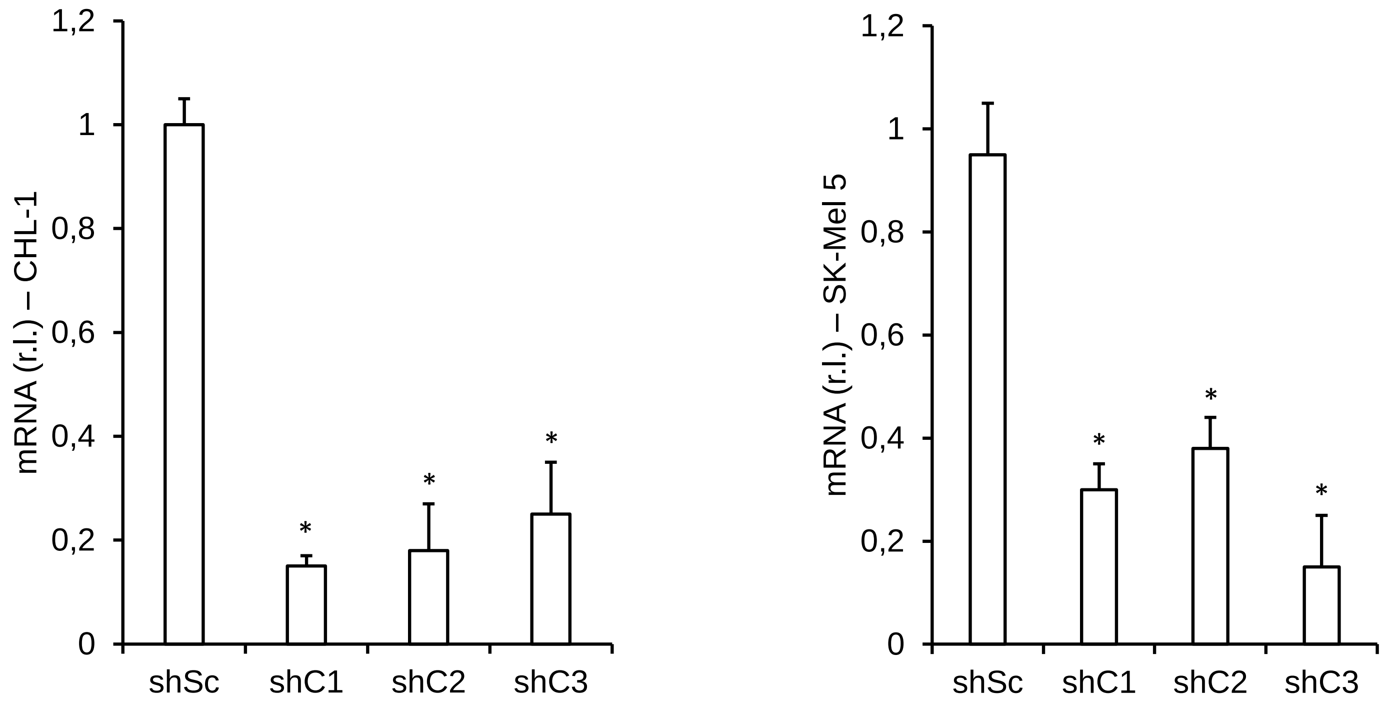

Supplementary Figure S6. AKRs expression inhibition. The expression of AKR1C1 (shC1), AKR1C2 (shC2) or AKR1C3 (shC3) was inhibited by infecting CHL-1 or SK-Mel 5 cells with lentiviral particle carrying specific shRNA (a scrambled sequence was used as control, shSc), and the expression of each AKRs was evaluated by qRT-PCR, 48h post-infection. (Histograms are representative of mean ± s.d.; n = 3).

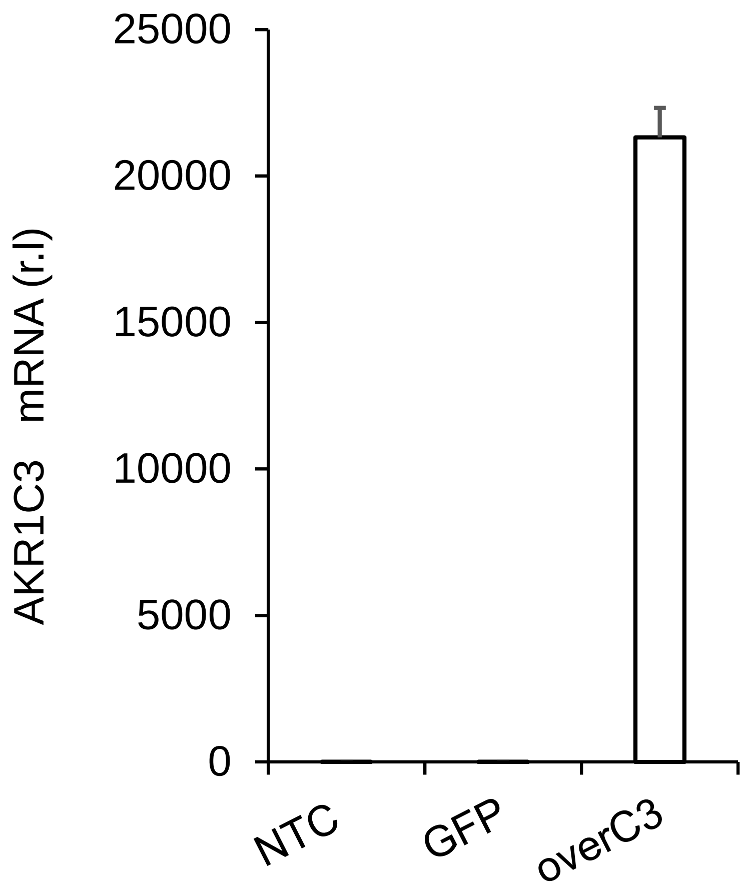

Supplementary Figure S7. AKRs confer resistance to ferroptosis execution. A2058 cells were not transfected (NTC) or transiently transfected with a vector coding for human AKR1C3, or a GFP encoding vector (used as control). The expression of AKR1C3 was evaluated by qRT-PCR.

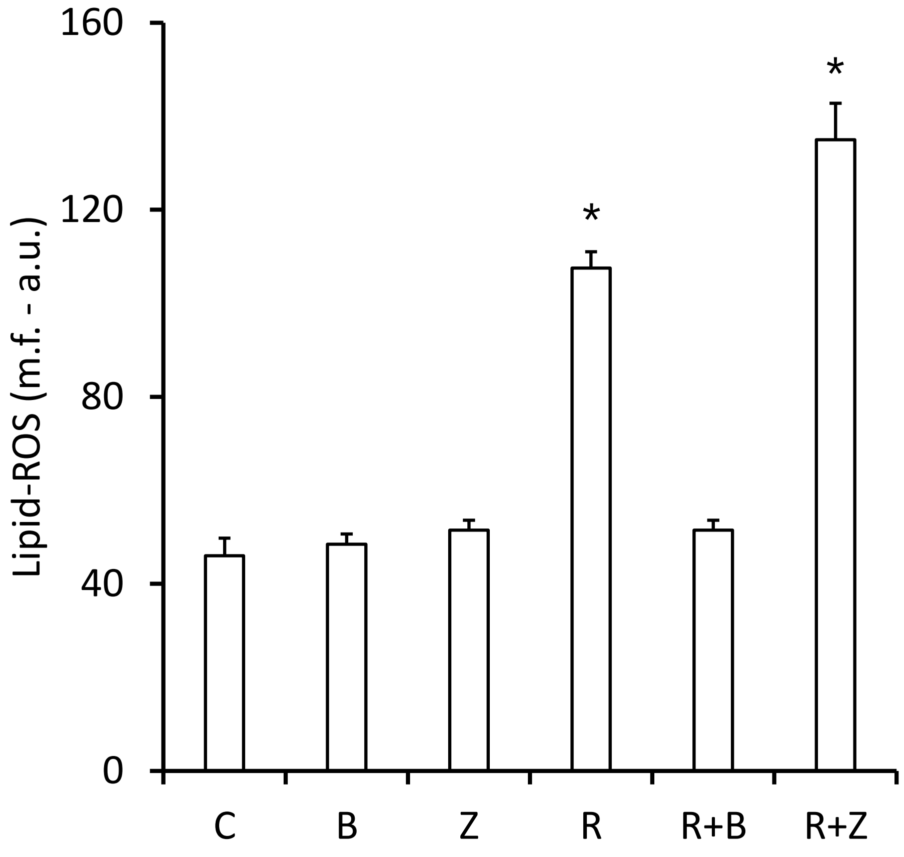

Supplementary Figure S8. Baicalein but not Zileuton blocks RSL3-induced lipid peroxidation. A375 cells were untreated (C) or treated with Baicalein (B, 20μM), Zileuton (10μM) RLS3 (2μM), RSL3 plus Baicalein (R+B) or RLS3 plus Zileuton, and lipid-ROS generation was evaluated 16h post treatment. (Histograms are representative of mean ± s.d.; n = 3).

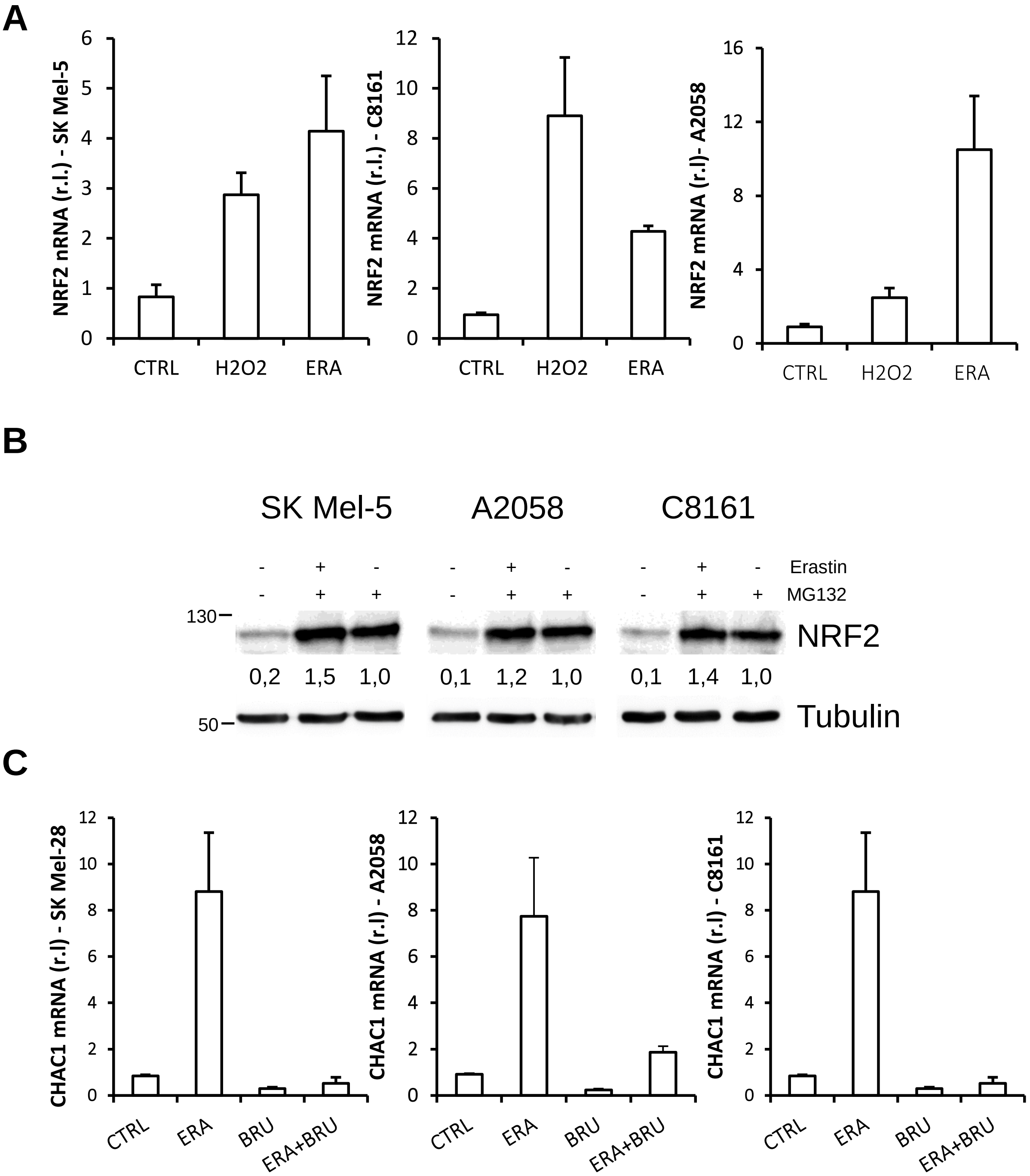

Supplementary S9. NRF2 and Ferroptosis. (A) The indicated cell lines were treated or untreated 4h with 10µM erastin or 500µM H_2O_2 and NRF2 mRNA levels were evaluated by qRT-PCR. (B) NRF2 protein levels were measured in cells exposed 4h to 10µM erastin in presence or absence of 10µM MG132 (2h pre-treatment). Tubulin was used as loading control. Densitometric analysis was reported. (C) The same cell line used in B were exposed 4h to 10µM erastin, 50nM Brusatol individually or concomitantly (ERA+BRU) and CHAC1 mRNA levels were evaluated by qRT-PCR. (Histograms represent mean±s.d.; n = 3; *p<0,05; w.b. images are representative of three independent experiments).

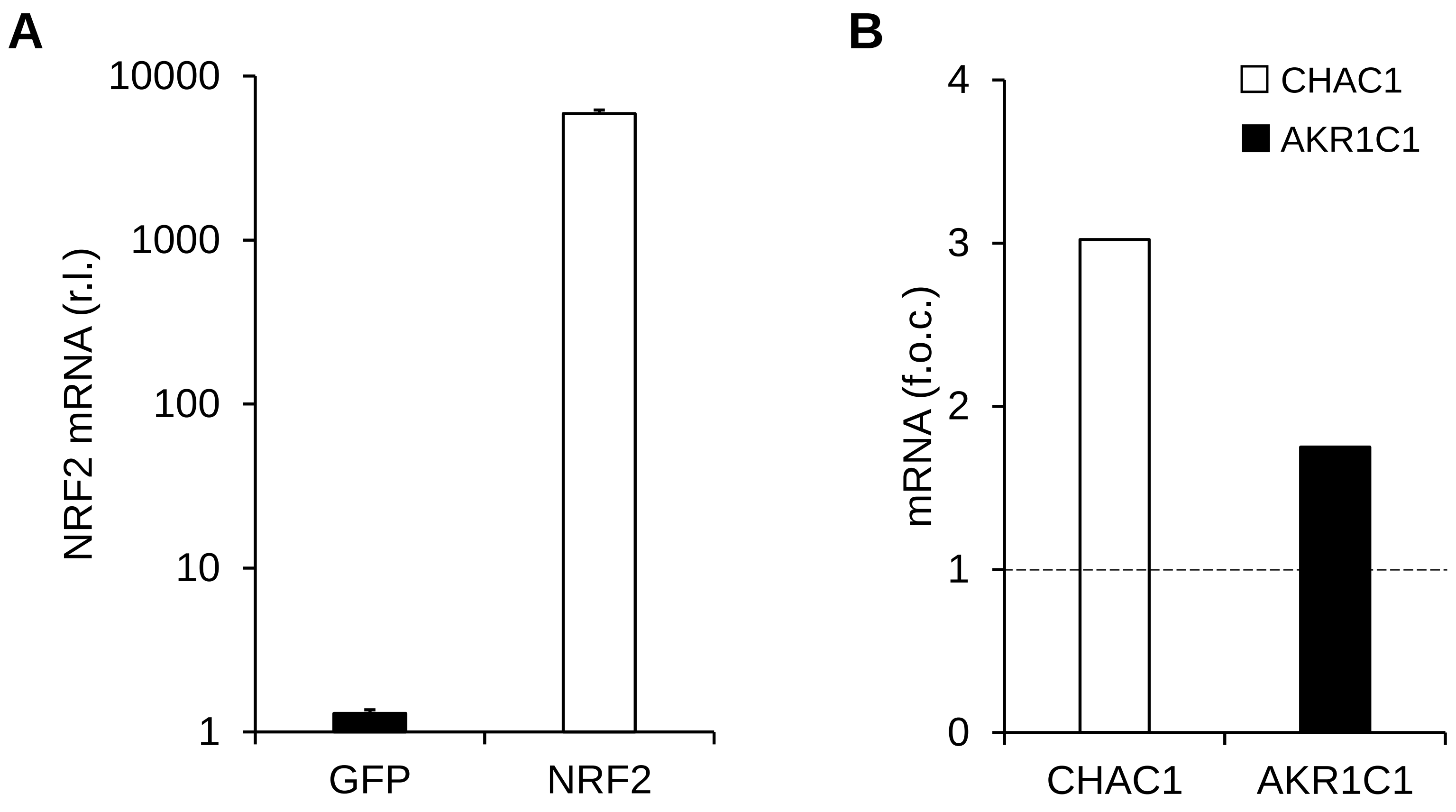

Supplementary Figure S10. NRF2 regulates CHAC1 and AKR1Cs during ferroptosis. NRF2 was (transiently) ectopically expressed in CHL-1 cells (A) and CHAC1 or AKR1C1 (B) expression was evaluated by qRT-PCR.

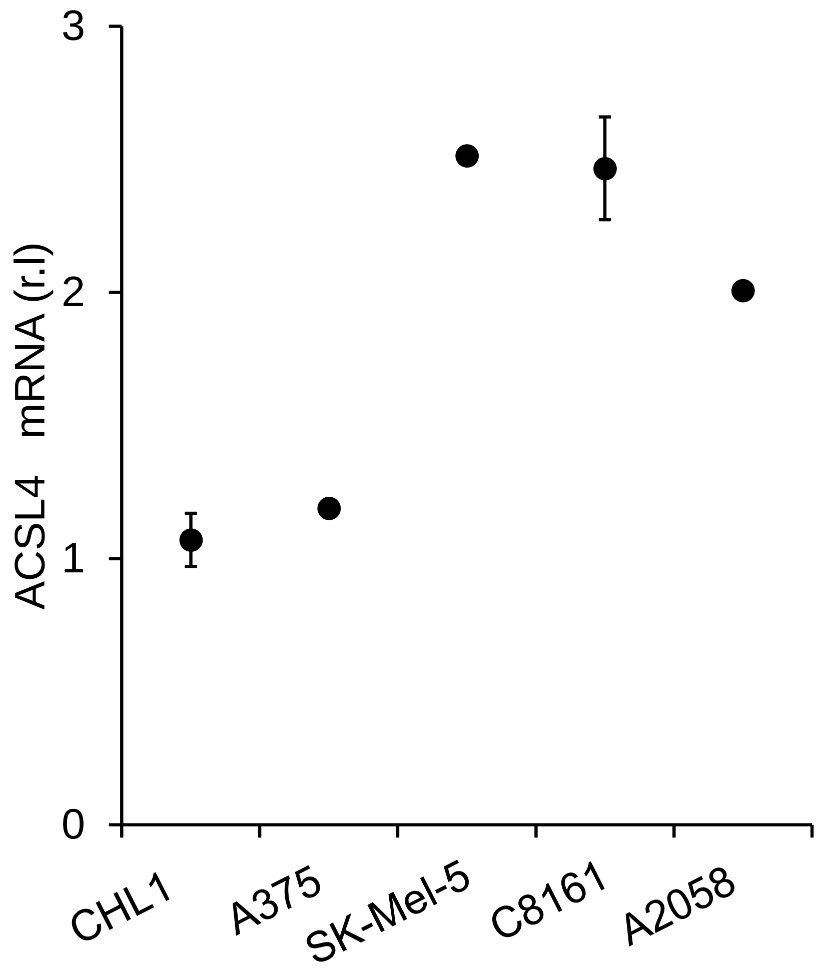

Supplementary Figure 11. ACSL4 expression and ferroptosis sensitivity of melanoma cells. (A) Basal expression of ACSL4 was evaluated in the indicated cell lines by qPCR (N=3; each point represents mean±s.d.)

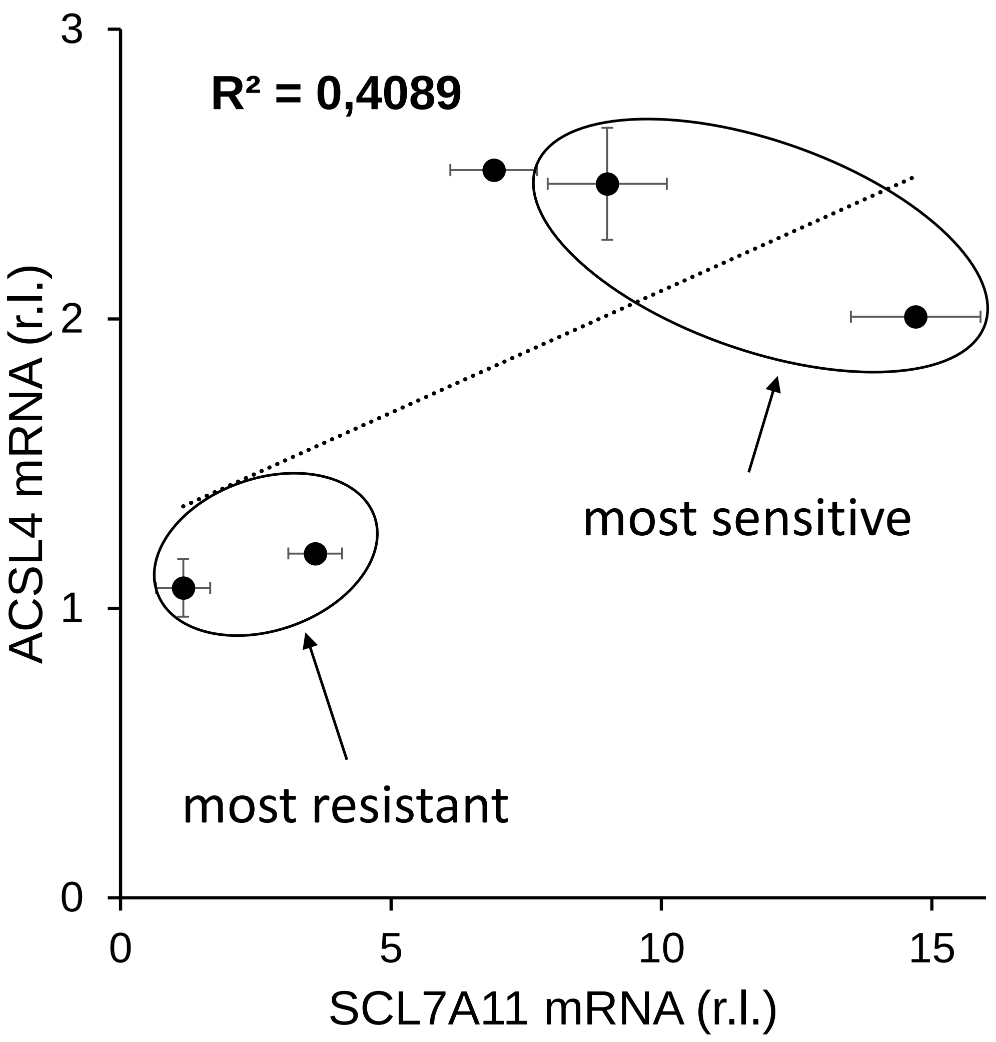

Supplementary Figure 12. ACSL4 vs SCL7A11 in melanoma cells. Basal expression of ACSL4 (Suppl. S10) was compared to basal SLC7A11 expression (Fig.5B) in each cell line (CHL-1, A375, SK-Mel 5, C8161 and A2058). The correlation coefficient R^2 was calculated, and the most ferroptosis sensitive and resistant cell lines were highlighted.

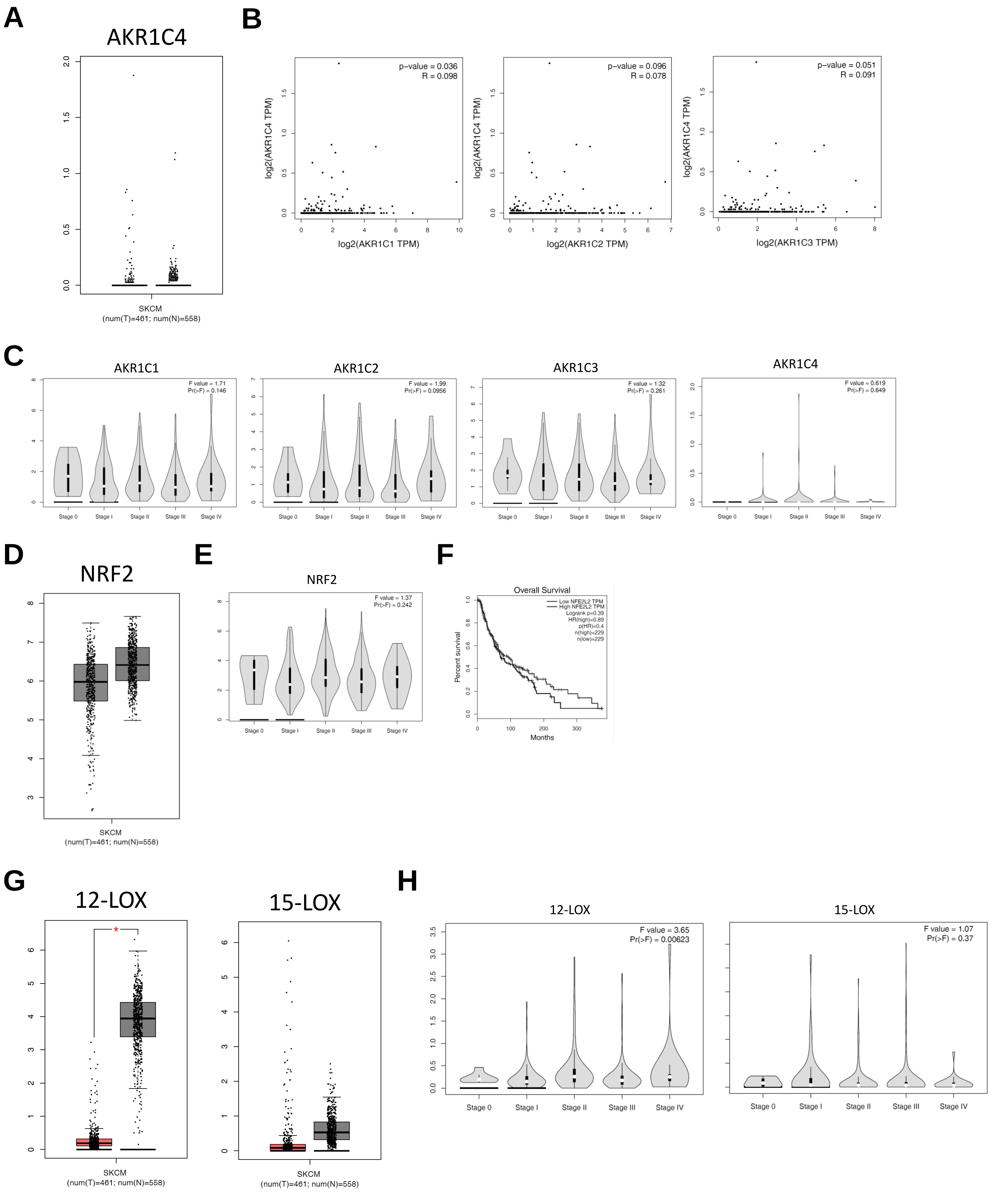

Supplementary Figure S13. AKR1C4 and ALOX comparative expression in melanoma vs normal tissues. A comparative analysis of AKR1C4 (A), NRF2 (D), 12-LOX (G, left) or 15-LOX (G, right) expression between melanoma and normal tissues was carried out by using the GEPIA free online platform (http://gepia.cancer-pku.cn/index.html). The total number of samples analysed was indicated under each boxplot. Analysis was performed setting the |Log2FC| Cutoff at 1 and a p-value Cutoff at 0,01. A correlative analysis between AKR1C4 and AKR1C1 (B, left panel), AKR1C2 (B, middle panel) or AKR1C3 (B, right panel) in tumor samples are also shown. The expression of AKRs (C), NRF2 (E) LOXs (H) were also evaluated during melanoma stage progression, as indicated. The overall survival was evaluated in both high and low expressing LOXs patients (F).
